# Neuroinformatic analyses of common and distinct genetic components associated with major neuropsychiatric disorders

**DOI:** 10.3389/fnins.2014.00331

**Published:** 2014-11-06

**Authors:** Amit Lotan, Michaela Fenckova, Janita Bralten, Aet Alttoa, Luanna Dixson, Robert W. Williams, Monique van der Voet

**Affiliations:** ^1^Department of Adult Psychiatry and the Biological Psychiatry Laboratory, Hadassah-Hebrew University Medical CenterJerusalem, Israel; ^2^Department of Human Genetics, Donders Institute for Brain, Cognition and Behaviour, Radboud University Medical CenterNijmegen, Netherlands; ^3^Department of Cognitive Neuroscience, Donders Institute for Brain, Cognition and Behaviour, Radboud University Medical CenterNijmegen, Netherlands; ^4^Department of Psychiatry, Psychotherapy and Psychosomatics, Psychiatric Neurobiology Program, University of WürzburgWürzburg, Germany; ^5^Department of Psychiatry and Psychotherapy, Medical Faculty Mannheim, Central Institute of Mental Health, University of HeidelbergMannheim, Germany; ^6^Department of Genetics, Genomics and Informatics, Center for Integrative and Translational Genomics, University of Tennessee Health Science CenterMemphis, TN, USA

**Keywords:** major neuropsychiatric disorders, neuroinformatics, cross-species, translational, genetic components, genome wide association studies, enrichment

## Abstract

Major neuropsychiatric disorders are highly heritable, with mounting evidence suggesting that these disorders share overlapping sets of molecular and cellular underpinnings. In the current article we systematically test the degree of genetic commonality across six major neuropsychiatric disorders—attention deficit hyperactivity disorder (ADHD), anxiety disorders (Anx), autistic spectrum disorders (ASD), bipolar disorder (BD), major depressive disorder (MDD), and schizophrenia (SCZ). We curated a well-vetted list of genes based on large-scale human genetic studies based on the NHGRI catalog of published genome-wide association studies (GWAS). A total of 180 genes were accepted into the analysis on the basis of low but liberal GWAS *p*-values (<10^−5^). 22% of genes overlapped two or more disorders. The most widely shared subset of genes—common to five of six disorders–included *ANK3, AS3MT, CACNA1C, CACNB2, CNNM2, CSMD1, DPCR1, ITIH3, NT5C2, PPP1R11, SYNE1, TCF4, TENM4, TRIM26*, and *ZNRD1*. Using a suite of neuroinformatic resources, we showed that many of the shared genes are implicated in the postsynaptic density (PSD), expressed in immune tissues and co-expressed in developing human brain. Using a translational cross-species approach, we detected two distinct genetic components that were both shared by each of the six disorders; the 1st component is involved in CNS development, neural projections and synaptic transmission, while the 2nd is implicated in various cytoplasmic organelles and cellular processes. Combined, these genetic components account for 20–30% of the genetic load. The remaining risk is conferred by distinct, disorder-specific variants. Our systematic comparative analysis of shared and unique genetic factors highlights key gene sets and molecular processes that may ultimately translate into improved diagnosis and treatment of these debilitating disorders.

## Introduction

Common psychiatric disorders including attention-deficit/hyperactivity disorder (ADHD), anxiety disorders (Anx), autism spectrum disorder (ASD), bipolar disorder (BD), major depressive disorder (MDD), and schizophrenia (SCZ), have a strong heritable component. Estimates for ASD range as high as 80% whereas those for anxiety range, 30–45% (Stein et al., 1999; Hettema et al., 2001). There is mounting evidence that many of these pervasive neuropsychiatric disorders share partially overlapping sets of common genetic risk factors (Cross-Disorder Group of the Psychiatric Genomics Consortium, 2013), supported by diagnostic comorbidities and shared phenotypes. Diagnostic comorbidities are often reported in psychiatric disorders (e.g., ADHD together with autism Joshi et al., 2014), depression and anxiety (Hamilton et al., 2014), and BD with ADHD (Faraone et al., 2012) and phenotypes shared among these disorders include deficits in sensorimotor gating, sleep, cognition, and social interaction (Braff et al., 2001; Spiegelhalder et al., 2013; Foussias et al., 2014).

The correct identification of both common and disease-specific sets of genes that confer higher or lower risk of developing these disorders should expose mechanisms of comorbidity and provide valuable targets for therapeutic intervention or prevention. However, the discrete (as opposed to dimensional) diagnostic system currently in use by mental health professionals (American Psychiatric Association: Diagnostic and Statistical Manual of Mental Disorders, Fifth Edition, DSM-5, 2013) is heavily criticized for its lack of low validity in terms of biological underpinnings (construct validity) as well as treatment response (predictive validity; Insel, 2014). Therefore, in line with the National Institute of Mental Health (NIMH) recently-launched Research Domain Criteria (RDoC) project (www.nimh.nih.gov/research-priorities/rdoc/index.shtml; Insel, 2014), the genetic dissection of molecular and cellular mechanisms underlying the different major disorders could provide complementary insight into the etiology and pathogenesis.

Using the National Human Genome Research Institute (NHGRI) catalog as a primary resource, we collected the set of genes that have been associated with six major categories of neuropsychiatric disorders [single-nucleotide polymorphisms (SNPs) with a *p*-value < 1.0 × 10^−5^]: ADHD, Anx, ASD, BD, MDD, and SCZ. To avoid biases caused by unequal sizes of gene sets, we restricted ourselves to analyzing similarly sized gene sets, resulting in top-51 protein-coding genes for each disorder. We refer to this well-vetted list as the NHGRI-cross-disorder gene set.

Given the (inherently) rigid boundaries of these symptom-based categories that are often poorly correlated with other relevant cognitive, imaging, and physiological abnormalities, we decided to pool GWAS results within each one of these six categories. For instance, GWAS findings from individuals with SCZ and schizoaffective disorders were pooled under the SCZ category. With respect to anxiety disorders, recent studies of people with a range of anxiety disorders demonstrate that those with a diagnosis of a specific anxiety disorder, such as posttraumatic stress disorder, may be at opposite extremes for startle reactivity, suggesting two biologically different disorders sharing the same diagnosis (Mcteague and Lang, 2012). On the other hand, recent studies emphasized commonalities among supposedly distinct anxiety disorders, thus highlighting the validity of a transdiagnostic approach toward anxiety disorders. Therefore, GWASs targeting individuals across a range of anxiety disorders were pooled under a category named anxiety disorders (Spielberg et al., 2014).

In the current analysis we use bioinformatic and analytic approaches, including molecular cohesivity, expression, and cross-species phenotype analysis with respect to brain regions involved in the pathogenesis of the disorders. We investigated (1) protein-protein interactions using data from curated databases. These represent a valuable resource of information on functions shared between genes (Lage, 2014), membership of a set of genes in a common pathway is often assumed based on interactions between their products (Segal et al., 2003). We further examined (2) enrichment of NHGRI-cross-disorder gene products in available proteomes of neuronal compartments. We focused in particular on human postsynaptic density (PSD), because many of the proteins within the PSD are important for neuronal functioning. They are enriched in cognitive phenotypes and cause neurological disorders (Bayes et al., 2011). The genes identified in SCZ-associated copy number variations (CNVs) have already been shown to have significant enrichment in the PSD proteome (Kirov et al., 2012). The relations between genes are also often represented by similar phenotypes that arise when they are disrupted. Therefore, we explored (3) the most prominent phenotypes associated to orthologs of NHGRI-cross-disorder genes in cross-species phenotype database (Uberpheno; Kohler et al., 2013). Next, we looked into (4) information on tissue with the highest expression for each gene set and identified co-expressed modules across gene sets during development of the most enriched tissue, the brain.

The final aim of our work was to dissect the genetic makeup of these six major neuropsychiatric disorders into Principal Components (PCs) based on co-expression patterns across different mouse strains, in an attempt to reveal genetic (and biological) mechanisms shared across all disorders. First, as a prerequisite for the next stage, we attempted to identify statistical correlations between such synthetic genetic components and relevant behavioral phenotypes (5). As this attempt was essentially intended to support the validity of a translational cross-species approach for use in the next stage, we limited this proof-of-concept analysis to two disorders. Since estimated heritabilities of anxiety disorders are in the modest range, 30–40%, significantly lower than for disorders such as SCZ (Hettema et al., 2001), these two disorders were chosen a priori since they represent markedly different diagnostic categories in terms of their relative heritability. In the next stage, we attempted to reveal whether distinct genetic components shared by all six disorders exist, and if so hypothesize about their postulated biological functions. For this analysis the mouse amygdala was chosen as our region of interest, since both structural and functional changes in this region have been consistently associated with anxiety disorders (Rauch et al., 2003), mood disorders (MDDs and BD, (Price and Drevets, 2010) SCZ (Benes, 2010), and ASD (Dziobek et al., 2010).

## Materials and methods

### Genes associated with major neuropsychiatric disorders

Genes associated with major neuropsychiatric disorders were mined from the NHGRI catalog of published genome-wide association studies (GWAS; Welter et al., 2014). The full catalog was downloaded (Hindorff et al., 2014) and publications were filtered on the keywords for neuropsychiatric disorders: Asperger disorder, attention deficit hyperactivity disorder, autism, BD, depression, depressive disorder, mood disorder, neuroticism, panic disorder, and SCZ. The published GWAS SNPs within 10 kb of a genomic feature (i.e., gene/transcript biotypes, see below) were selected and duplicate genomic features within each disorder were filtered out. Protein-coding genomic features were used for further analysis, which were retrieved through filtering using the Ensembl BioMart feature Biotype and manual curation for unannotated features. To limit discovery bias, same-sized sets of features were extracted for subsequent analysis (*n* = 55), from which 20 non-coding features were excluded, namely: antisense (2x), lincRNA (6x), ncRNA (5x), pseudogene (6x), rRNA (1x). This resulted in same-sized coding gene sets (*n* = 51) with the exception of anxiety (*n* = 16), due to limited association of genes.

### Annotation of mouse orthologs

Mouse homologs of the human genes were retrieved through a BioMart query using Ensembl Compara ortholog prediction (Vilella et al., 2009).

### Venn diagram

The overlap between neuropsychiatric disorders was visualized using a Venn diagram, created using a webtool: (http://bioinformatics.psb.ugent.be/software/details/Venn-Diagrams). Graphical output is only possible for up to five lists (ADHD, ASD, BD, MDD, SCZ). The two overlapping Anx genes were annotated manually.

### Protein-protein interactions

Cytoscape (version 2.8.3) was used to visualize protein-protein interaction networks based on curated interactions from the BioGRID protein-protein interaction online data repository (Release 3.2.108) and HPRD protein Reference Database (Release 9). A background interaction set created by merging BioGRID and HPRD data was further adjusted by removing duplicates and non-physical interactions. The resulting set contained purely physical interactions (i.e., association in complex, direct interaction, physical interaction, and biochemical co-localization).

### Gene ontology

GOrilla (cbl-gorilla.cs.technion.ac.il; Eden et al., 2009), DAVID (http://david.abcc.ncifcrf.gov/; Huang Da et al., 2009a,b) and WebGestalt (http://bioinfo.vanderbilt.edu/webgestalt/; Zhang et al., 2005; Wang et al., 2013) tools were used for gene ontology (GO) annotation.

### Enrichment analysis

Data sets used for calculation of enrichment: human postsynaptic density proteins (hPSD; Bayes et al., 2011), nuclear proteome isolated from human brain (Dammer et al., 2013), membrane-enriched proteome from human brain (Donovan et al., 2012), mitochondrial proteome from mouse brain (Stauch et al., 2014), proteome from rat synaptic vesicles (Morciano et al., 2005), cross-species phenotype ontology (Uberpheno; Kohler et al., 2013). Enrichment and *p*-value were calculated with two-sided Fisher exact test.

### Identification of tissue with highest expression of NHGRI-cross disorder gene sets

Data from Illumina Human BodyMap 2.0 (http://www.illumina.com/science/data_library.ilmn) summarized to tissue with highest expression for each annotated gene (*n* = 166), was used for tissue enrichment analysis. *p*-values were calculated using two-sided Fisher exact test.

### Co-expression and temporal specificity

Normalized gene-expression data determined by RNA sequencing and representing 16 human brain regions were obtained from BrainSpan (http://www.brainspan.org). Expression for 179 out of 186 NHGRI-cross-disorder genes was extracted and clustered according to their expression pattern with R-package WGCNA (weighted correlation network analysis, Langfelder and Horvath, 2008). The expression level for each gene and developmental stage (only stages with expression data for all structures were selected, *n* = 12) was calculated as median expression across all regions at a given stage.

### Establishing mouse ortholog probes corresponding to the NHGRI-cross-disorder gene sets and principal component analysis of co-expression patterns in mouse amygdala

For each gene in the NHGRI-cross-disorder gene set, an adequate probe within the well-curated INIA Amygdala Cohort Affy MoGene 1.0 ST (Mar11) RMA Database was identified using GeneNetwork (www.genenetwork.org). This set contains expression data from 54 genotypes of BXD mice, which were generated by crosses of C57BL/6J and DBA/2 inbred strains (Wang et al., 2003). The amygdala region was chosen based upon biological and practical considerations. If several probes for the same gene were available, the probe with the highest expression value was selected. Based upon co-expression patterns across the 50 BXD and parent strains, a Principal Component Analysis (PCA) was undertaken. For each disorder, the synthetic PCs that individually account for >10% of the total variance in the probe set expression were identified.

### Expression–phenotype correlations

As outlined in the introduction, this prerequisite feasibility analysis was limited to SCZ and anxiety disorders. For each category, well-established behavioral paradigms for which consistent ethological data from rodents suggests face and predictive validities to the corresponding disorders in humans have been selected for correlation with the PCs derived above. Subsequently, based on classical approach-avoidance paradigms (Cryan and Sweeney, 2011), the two anxiety-related mouse phenotypes selected were the elevated plus maze and dark-light box traits reported by Yang et al. (2008). For SCZ-related mouse phenotypes, based on sensorimotor gating paradigms that apparently reflect an interface between psychosis and cognition (van Den Buuse, 2010), we selected behavioral traits obtained from experiments measuring the prepulse inhibition of acoustic startle response reported by Loos et al. (2012). For each disorder, we performed a PCA of the relevant behavioral traits. Individual PCs accounting for >10% of total variance was then cross-correlated with the disorder-specific expression PCs (see above). Pearson's moment product or Spearman's rank-order correlations were used, depending on the number of subjects per group, trait distribution and existence of outliers. For each disorder, correlations were deemed significant at *p*-values < 0.05 following Bonferroni's correction for multiple testing.

### Cross-species analysis of genetic components shared across disorders

A second-order PCA among all of the disorder-specific amygdalar co-expression PCs generated previously was performed, in an attempt to reveal if the new second-order PCs—termed Master PCs—could be detected and whether they receive significant contribution from all six neuropsychiatric disorders.

### Biological underpinning of the shared genetic components—translational evidence from mice

In order to attribute a biological meaning to shared genetic components, if indeed identified, for each Master PC, a list of the top-500 genes with the highest genetic correlation across all 34760 records of the Amygdala Cohort Database was assembled. Enrichment analysis based on GO terms was then performed for each list using WebGestalt (Zhang et al., 2005; Wang et al., 2013). The mmusculus__genome was chosen as the reference gene set. For the statistical analysis, the hypergeometric method was chosen. Multiple test adjustment was performed using the BH method (Benjamini and Hochberg, 1995). Minimum number of genes for a category was set at two. Significantly enriched GO terms (i.e., those with adjusted *p* < 0.05) were presented using REVIGO (Supek et al., 2011).

## Results

### Curating the disorder-specific gene sets based on current GWASs

**Figure 1:**
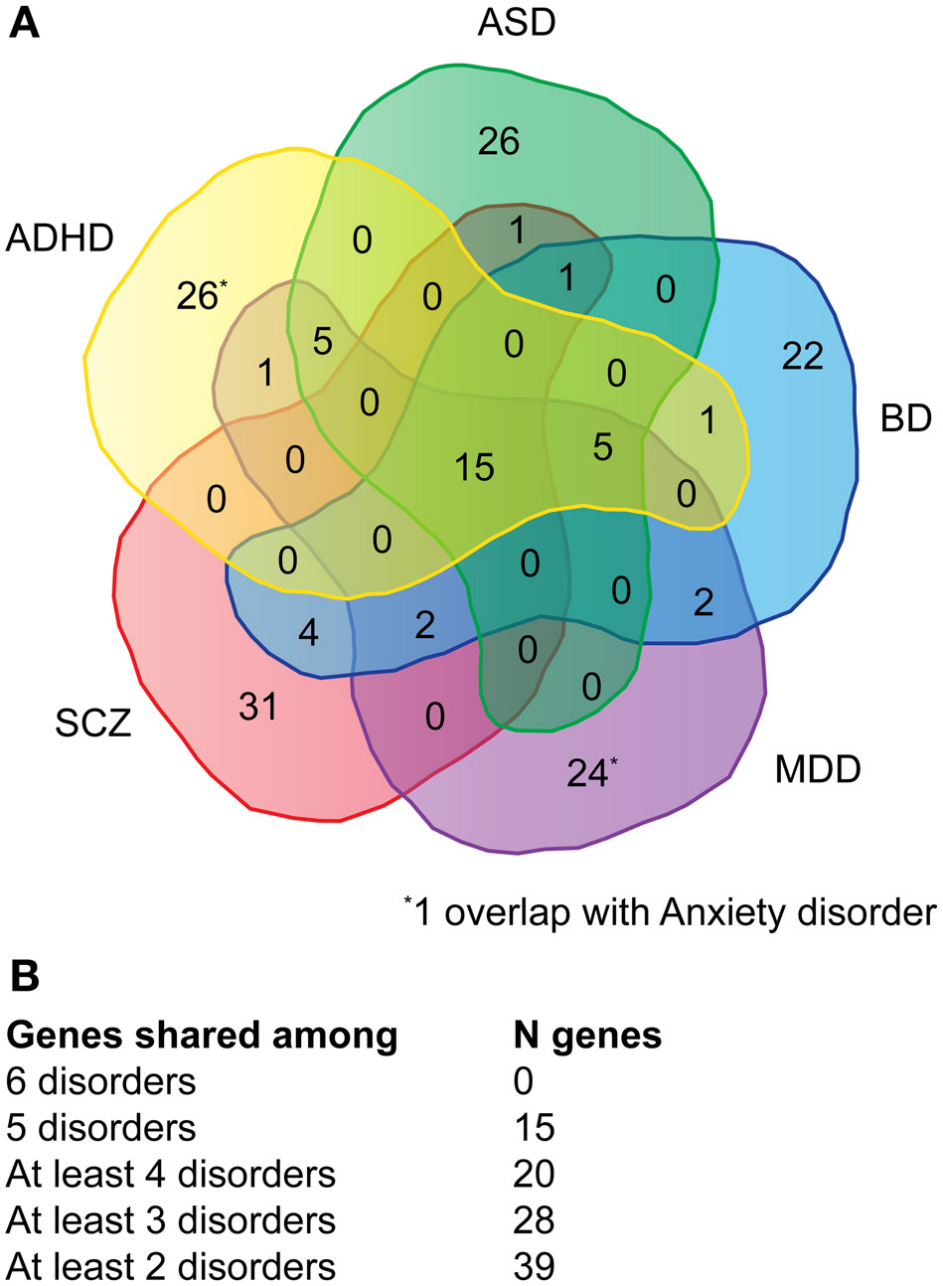
**Venn diagram depicting the overlap of genes across multiple disorders. (A)** For each disorder (ADHD, ASD, BD, MDD, SCZ) the overlap of top-51 SNPs with associated protein-coding genes is depicted. For anxiety only 16 protein-coding genes could be retrieved, one overlaps with ADHD and one with MDD. **(B)** Summary of the number of genes shared among disorder. A detailed list of genes can be viewed in Supplementary Table 1.

Genes associated with major neuropsychiatric disorders were mined from the NHGRI catalog of published GWAS (Welter et al., 2014). A total of 115 publications (Supplementary Table 1A) were retrieved reporting 911 SNPs with a *p*-value < 1.0 x 10^−5^. The top-51 protein-coding genes were selected for each disorder, with the exception of anxiety where only 16 genes could be retrieved (Supplementary Table 1B). Of the 180 genes (referred as NHGRI-cross-disorder gene set throughout the manuscript), 15 occurred in at least five disorders (8%), 20 occurred in at least four disorders (11%), 28 occurred in at least three disorders (16%), 39 occurred in at least two disorders (22%) (Figures 1A,B).

### The NHGRI-cross-disorder gene set does not form interconnected protein-protein interaction network and does not split into go enriched categories

With focus on identification of common biological processes that potentially control disease pathogenesis, we investigated protein-protein interactions within the NHGRI-cross-disorder gene set. Contrary to our prediction, when mapping the protein-coding genes from NHGRI-cross-disorder set to a merged and curated BioGRID and HPRD protein-protein interaction database (containing only physical interactions, see Material and Methods), only 152 proteins and nine interactions were found (Supplementary Figure 1). The NHGRI-cross-disorder set does thus not represent an interconnected network on the level of available direct protein-protein interactions.

Despite a lack of direct interactions, core proteins might be connected via second interactors that have not yet been identified in disease-association studies, but might represent molecular nodes in functional networks. We therefore created a network from NHGRI-cross-disorder proteins occurring in at least five disorders (the most shared subset of proteins) and allowed novel interactors to connect at least two query proteins. Twelve of 15 proteins were connected by six interactors (Supplementary Figure 2). These interactors were proteins involved in ubiquitination (UBE2I, UBC, CAND1), intracellular Ca^2+^ signaling (CALM1) and intracellular cAMP signaling (PRKACA, EP300). Two interactors have been associated with Alzheimer's disease (CAND1, UBE2I), but none have yet been associated with neuropsychiatric disorders. The core interactor, UBC (Ubiquitin C), is a protein with large number of natural binding partners (*n* = 8750) thus interaction is unlikely to be specific for psychiatric disorders. We consider this result less informative since we have not tested significance of this network over random sets.

GO enrichment analysis of 15 genes present in at least five disorders did not highlight any enriched categories, neither did the GO analysis of the complete NHGRI-cross-disorder gene set. These results complicate conclusions on functional relatedness of studied genes.

### NHGRI-cross-disorder genes are enriched in the human postsynaptic density and are linked to similar phenotypes

The top-51 genes associated with major neuropsychiatric disorders discovered by GWAS do not interact on the protein level and (as a whole) do not show enrichment in GO categories. To address the functional relatedness by other means, enrichment of protein localization in neuronal subcellular structures was performed. Comprehensive and systematic characterization of neuronal proteome is not yet available but techniques for isolation of sub-neuronal compartments and identification of protein content have improved and proteome data from some organelles/sub-structures such as nucleus (Dammer et al., 2013), membrane (Donovan et al., 2012), mitochondria (Stauch et al., 2014), synaptosome (Morciano et al., 2005) and PSD (Bayes et al., 2011) are available.

Enrichment analysis of the NHGRI-cross-disorder gene set in neuronal nucleus, membrane, mitochondria and synaptic vesicles showed depletion (in fact, membrane, mitochondria and synaptic vesicles did not contain any proteins from the NHGRI-cross-disorder set). Human PSD resulted in significant enrichment (score = 1.63; *p* = 2.09 x 10^−3^), confirming contribution of PSD components to pathology of major neuropsychiatric disorders.

**Figure 2:**
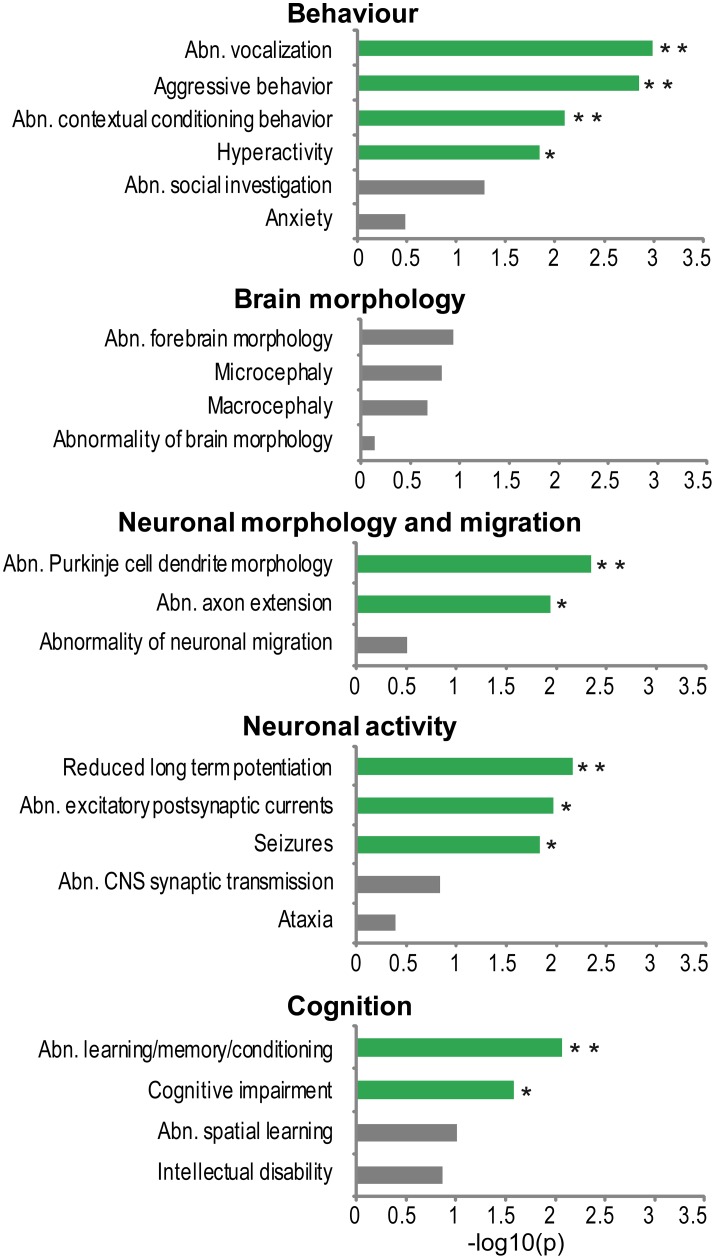
**Phenotype enrichment**. −log_10_(*p*-value) of cross-species phenotypes from Uberpheno grouped to broader categories. Significant terms are depicted in green; ^**^p < 0.01, ^*^p < 0.05.

To investigate what phenotypes in model organisms are most frequently associated to genes from the NHGRI-cross-disorder sets, enrichment of cross-species phenotypes based on phenotype ontology data (Uberpheno; Kohler et al., 2013) was analyzed. Associated phenotypes were ranked based on their frequency in the NHGRI-cross-disorder gene sets and investigated for enrichment of phenotype ontology terms relevant to neuropsychiatric disorders. For the complete list of phenotypes and frequencies, see Supplementary Table 2. The terms were grouped in five categories: (1) behavior, (2) brain morphology, (3) neuronal morphology and migration, (4) neuronal activity, and (5) cognition. Figure 2 depicts enrichment expressed as −log_10_(*p*) of all terms in these categories. Despite possible study bias, analysis of associated cross-species phenotypes revealed that there is phenotype cohesivity underlying genes associated to six major neuropsychiatric disorders.

The enrichment analysis suggests that rather than influencing brain structural malformations, the morphology of neurons (axons and dendrites) and synaptic transmission are important phenotypes. Some of the very specific behavioral phenotypes that are linked to NHGRI-cross-disorder genes are abnormal vocalization, aggressive behavior, abnormal contextual conditioning, and hyperactivity. Finally, cognitive impairment and abnormal learning/memory conditioning are also significantly enriched in the NHGRI-cross-disorder gene sets.

### Expression analysis—tissue specificity

**Figure 3:**
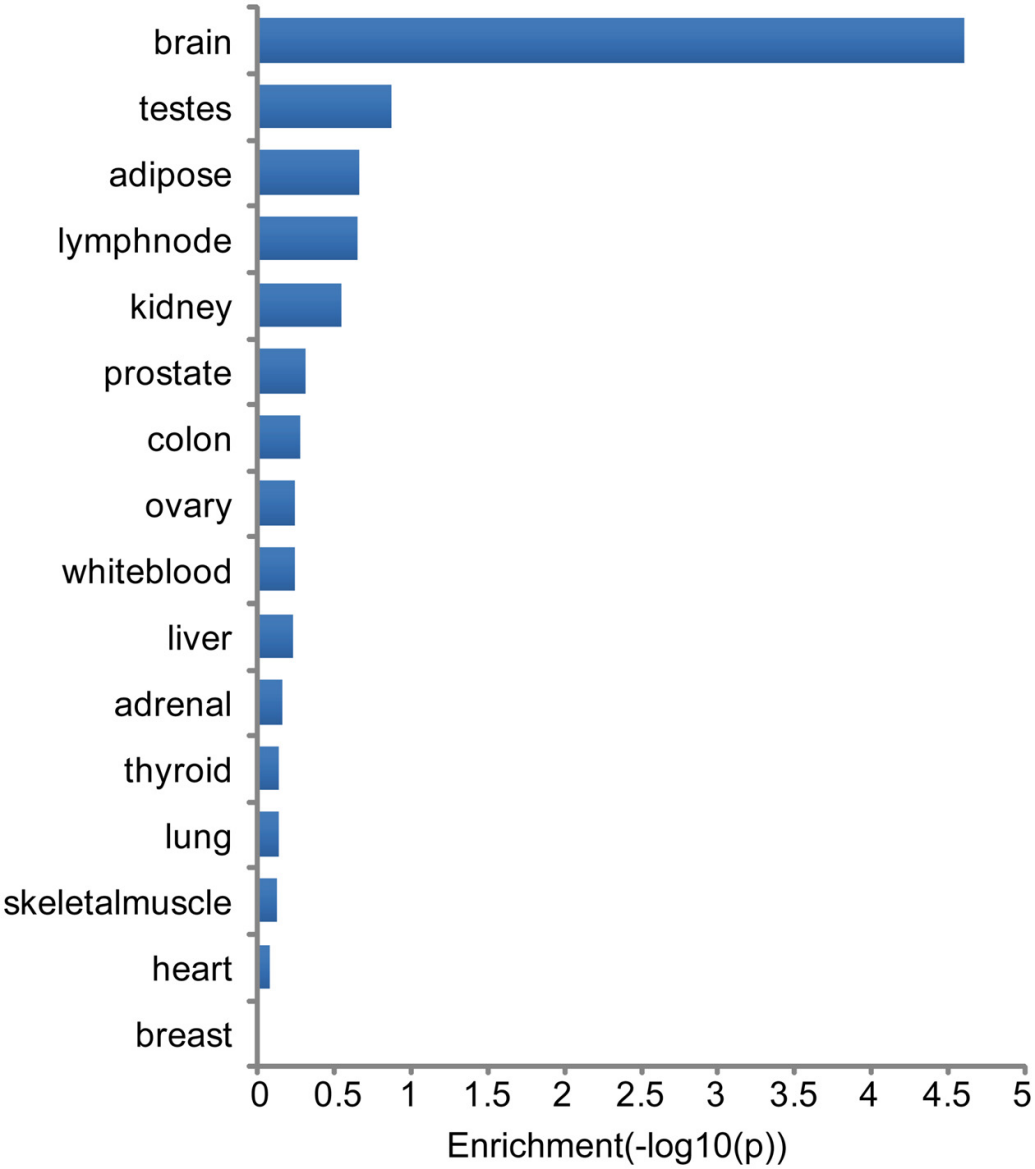
**Enrichment of tissues with highest expression in NHGRI-cross-disorder genes**. −log_10_(*p*-value) of enrichment of all tissues with highest expression level of NHGRI-cross-disorder genes. The most prominent tissue is brain with a significant enrichment of *p* = 2.42 x 10^−5^.

Tissue specificity of genes associated with major neuropsychiatric disorders was investigated through analysis of highest tissue expression of the NHGRI-cross-disorder gene sets. 166 genes from the NHGRI-cross-disorder gene set was mapped to Illumina Human BodyMap 2.0. A significant enrichment for highest expression was found in the brain (Figure 3; *p* = 2.42 x 10^−5^). Brain is the top tissue with highest gene expression for ADHD, Anx, and ASD. For BD, MDD, SCZ, brain is less prominent and occurs in the same frequency range as white blood, ovary, and testes. Table 1 summarizes top-4 tissues for each disorder.

**Table 1:** **Top-4 tissues with highest expression for each disorder**.

**Top-4 Tissues with highest expression**				
	**Tissue 1**	**Tissue 2**	**Tissue 3**	**Tissue 4**
All	Brain (37/166)	White blood (25/166)	Testes (23/166)	Ovary (15/166)
ADHD	Brain (12/48)	Testes (7/48)	White blood (6/48)	Ovary (5/48)
Anx	Brain (6/14)	Adipose (2/14)	Testes (1/14)	Skeletal muscle (1/14)
ASD	Brain (12/50)	White blood (7/50)	Testes (6/50)	Ovary (5/50)
BD	White blood (8/50)	Ovary (8/50)	Brain (7/50)	Testes (6/50)
MDD	White blood (9/49)	Brain (8/49)	Ovary (7/49)	Testes (6/49)
SCZ	White blood (9/49)	Testes (8/49)	Brain (6/49)	Liver (5/49)

### Genes from the NHGRI-cross-disorder set cluster in three co-expression modules with distinct spatio-temporal expression patterns and functional biases

**Figure 4:**
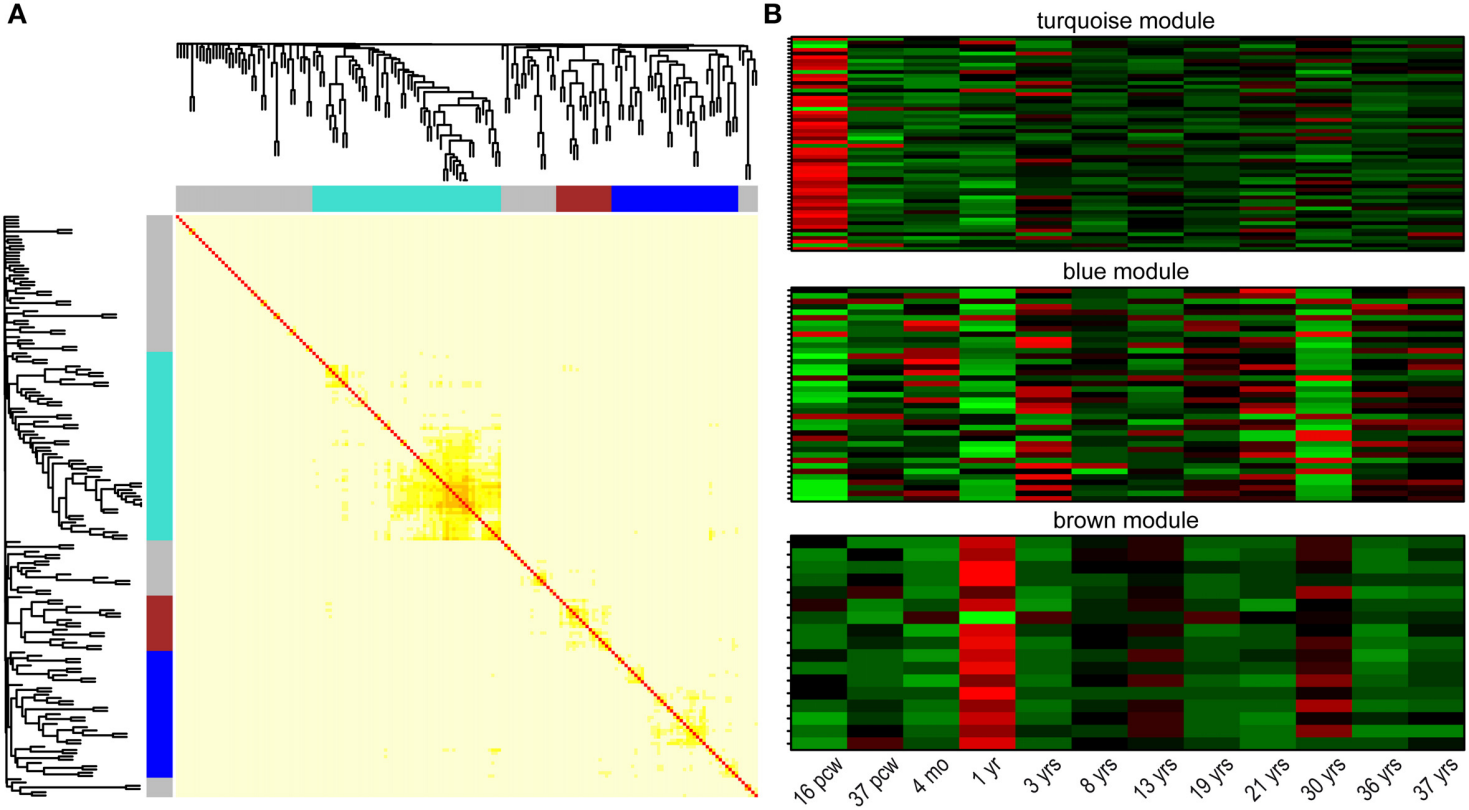
**Co-expression clusters among NHGRI-cross-disorder genes. (A)** Topological overlap matrix plot (TOMplot), a heatmap depicting the topological overlap matrix supplemented by hierarchical clustering dendrograms. Modules are depicted in colors (turquoise, blue, and brown). **(B)** Expression heatmap plots for each module. The following abbreviations are used: postconceptional weeks (pcw), months (mo), years (yrs).

One of the major properties of genes involved in regulation of common biological/cellular process—next to interactions on protein level—is their co-expression. Besides being the tissue with highest expression, the brain is logically the area of focus in search for gene co-expression networks in neuropsychiatric disorders. Correlation of gene expression was explored with the use of the recently released BrainSpan developmental transcriptome: mapping gene expression in 16 human brain structures across 26 developmental stages (Kang et al., 2011). Clustering 179 NHGRI-cross-disorder transcripts within a weighted gene co-expression network (using WGCNA; Langfelder and Horvath, 2008) in developing human brain revealed three gene modules with distinct spatiotemporal expression patterns (Figure 4). The turquoise module (*n* = 58 genes) is characterized by low expression during early fetal development (postconceptional week 16). The blue module (*n* = 39 genes) is characterized by high expression in early fetal development, early childhood (1 year), and at the age of 30 years. The brown module (*n* = 17 genes) is characterized by decreased expression and opposite peaks of expression at 1 and 30 years. Supplementary Table 3 contains the complete list of genes in all three modules and their connectivity. The modules do not follow clear and simple developmental expression trajectory, but they show sharp expression peaks, and troughs at specific developmental time points (postconceptional week 16, 1 year, 30 years). Disorder-specific gene enrichment analysis revealed that there is a particular contribution of disease-specific genes in the co-expression modules: BD genes are enriched in the blue module, Anx genes are enriched in the brown module, while SCZ genes are depleted in the brown module (Table 2 for significant enrichment, depletion and *p*-values; for complete enrichment data, see Supplementary Table 4). However, correlation of gene expression in the turquoise module did not show enrichment in any of the disorder-specific gene list and genes from this module are distributed throughout all lists. Biological processes controlled by these genes may represent connecting link between highly heterogeneous neuropsychiatric disorders.

**Table 2:** **Significant enrichments or depletions of disorder-specific genes in three co-expression modules identified by weighted correlation network analysis (WGCNA)**.

**Module**	**Disorder**	**Enrichment**	***p*-value**
Brown	Anx	3.13	4.98 x 10^−2^
Blue	BD	2.09	1.00 x 10^−2^
Brown	SCZ	0.15	2.57 x 10^−2^

Splitting of NHGRI-cross-disorder gene sets into tight co-expression modules turned out to be helpful in identification of genes with shared molecular functions. Follow-up GO enrichment analysis revealed distinct biological processes behind the three modules. The turquoise module was enriched for *regulation of gene expression* (GO:0010468; *p* = 2.62 x 10^−4^; FDR *q* = 7.88 x 10^−2^), *regulation of metabolic process* (GO:0019222; *p* = 7.15 x 10^−5^; FDR *q* = 2.87 x 10^−2^), and *protein binding* (GO:0005515; *p* = 4.77 x 10^−6^; FDR *q* = 1.31 x 10^−3^). The blue module was enriched for *neuron projection guidance* (GO:0097485; *p* = 8.13 x 10^−4^; FDR *q* = 6.56 x 10^−1^) and *axon guidance* (GO:0007411; *p* = 8.13 x 10^−4^; FDR *q* = 3.28 x 10^−1^), and the brown module is enriched for *response to stress* (GO:0006950; *p* = 4.2 x 10^−4^; FDR *q* = 2.41 x 10^−1^).

### Mouse orthologs of NHGRI-cross-disorder gene sets can be grouped in principal components based on their amygdalar co-expression patterns

Adequate probes for the vast majority of genes from the NHGRI-cross-disorder set were identified within the INIA Amygdala Cohort Affy MoGene 1.0 ST (Mar11) RMA Database (e.g., see Supplementary Tables 9,10 depicting probes for anxiety and SCZ-associated mouse orthologs, respectively). Following PCA, for each of the six disorders, two synthetic expression PCs individually accounting for >10% of total variance in the probe set expression were identified. Together, these PCs accounted for 30–45% of the total variance in expression of these probe sets (e.g., see Supplementary Figures 3, 4 depicting the scree plots for the anxiety and SCZ-associated mouse ortholog PCs). Altogether, 12 synthetic expression PC traits were created.

### Expression-phenotype correlations support the validity of the cross-species approach for analyzing the contribution of genetic components to neuropsychiatric disorders

**Figure 5:**
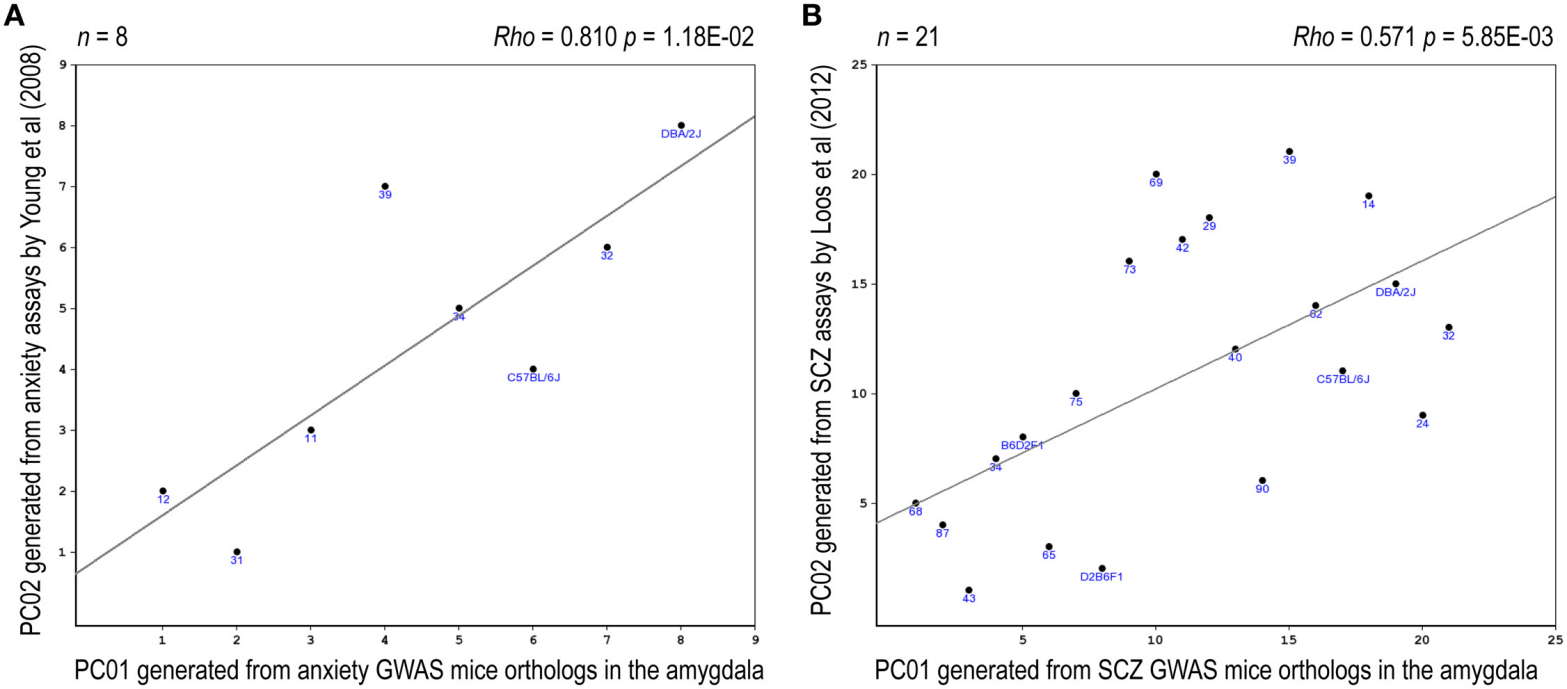
**Expression-phenotype correlations**. Correlations between the top-2 synthetic PCs derived from the expression profile of the mouse orthologs of the NHGRI-cross-disorder gene set and relevant mouse behavioral phenotypes are depicted. **(A)** Correlation between the top PCs derived from the expression profile of the anxiety probe set and the top PCs derived from anxiety-related behavioral traits. **(B)** Correlation between the top PCs derived from the expression profile of the SCZ probe set and the top PC derived from prepulse inhibition-related behavioral traits.

Eight relevant anxiety-related traits, based upon Yang et al. (2008), were identified in the BXD database (Supplementary Table 11). PCA revealed that the two top PCs together accounted for >80% in total variance of these anxiety traits (Supplementary Figure 5). A significant correlation between one of these anxiety-phenotype PCs and an anxiety-expression PC was noted (rho = 0.81, *p*_raw_ = 0.012, *p*_adj_ = 0.047, Figure 5A). Four relevant prepulse inhibition-related traits, based upon Loos et al. (2012), have been identified in the BXD database (Supplementary Table 12). PCA revealed that the top PC accounted for >90% in total variance of these prepulse inhibition traits (Supplementary Figure 6). A significant correlation between the top prepulse inhibition-related PC and an SCZ-expression PC was noted (rho = 0.57, *p*_raw_ = 0.006, *p*_adj_ = 0.012, Figure 5B).

### Genetic components are shared across all disorders

**Figure 6:**
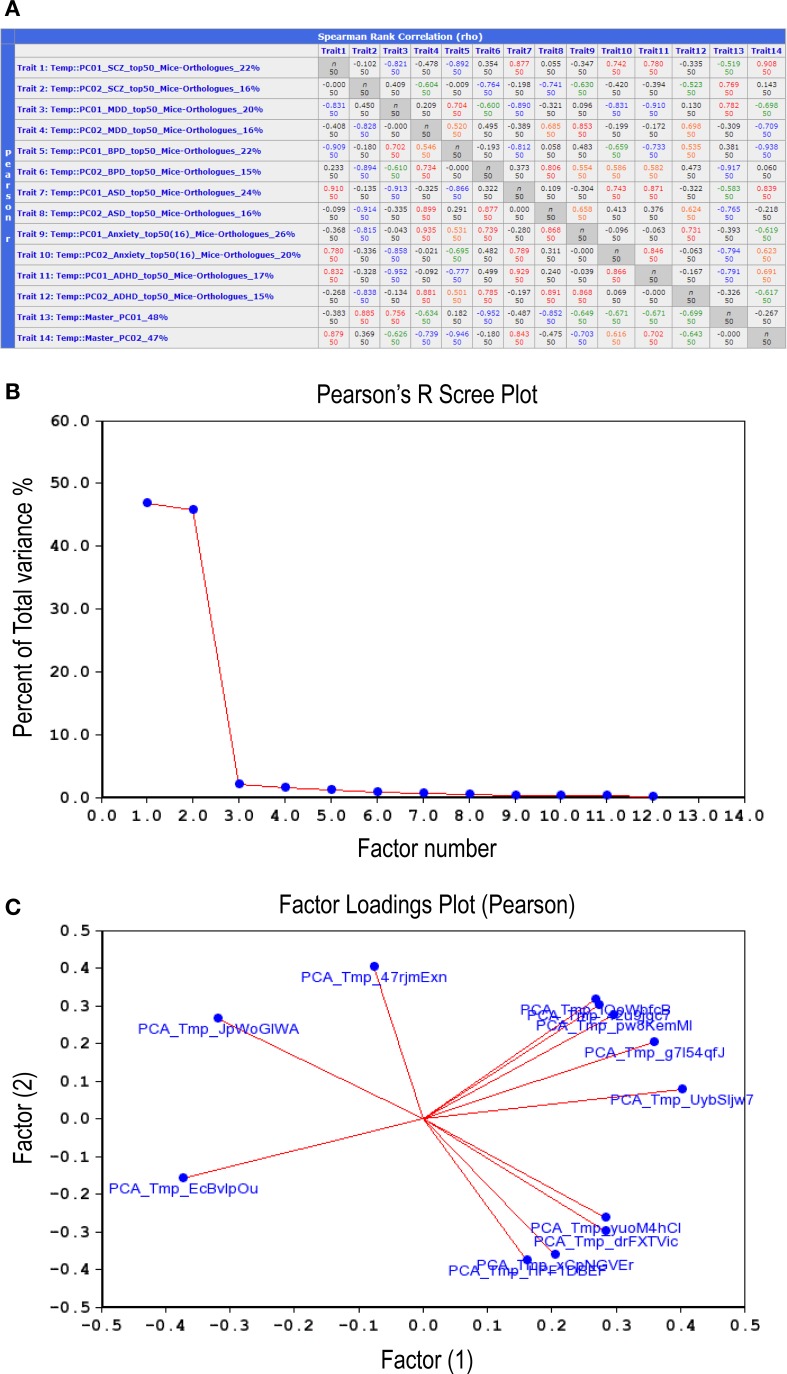
**Correlations between the top-12 synthetic PC traits. (A)** Second-order correlations between all 12 synthetic PC traits, two per disorder, are depicted. The number appearing at the end of each trait name (first column) denotes the percent of total variance for each PC generated from a disorder's top-51 gene-set mouse orthologs co-expression vector. The bottom two rows depict the second-order (termed Master) PCs that were generated by correlating the 12 synthetic PC traits amongst themselves. Lower left cells list Pearson product-moment correlations; upper right cells list Spearman rank order correlations. Each cell also contains the *n* of cases. Values higher than 0.7 are displayed in red; those between 0.7 and 0.5 in orange; between −0.5 and −0.7 in green; values lower than −0.7 are in blue. Scree plot **(B)** depicts the percentage of total variance across the 12 synthetic PC traits (>45%) that is accounted for by each one of the Master PCs. Factors loading plot **(C)** depicts the relative vector-wise contribution to both Master PCs arriving from each of the 12 disorder-specific PC traits.

Examination of correlations between the 12 synthetic expression PC traits described above (Figure 6A) suggested that they could be grouped into two distinct sets, or Master PCs, each accounting for nearly 50% of total variance (Figure 6B). A factor loading analysis revealed that 8–9 synthetic PC traits, coming from all six disorders, contributed to each of the Master PCs (Figure 6C, see also Figure 6A, bottom two rows). Thus, the two Master PCs synthesized from the combined list of individual disorders' top PCs are shared by all six neuropsychiatric disorders. Notably, these Master Traits most probably represent distinct co-expression vectors as they are poorly inter-correlated (rho = −0.27, *p* > 0.05).

### Biological underpinning of the shared genetic components—translational evidence from mice

**Figure 7:**
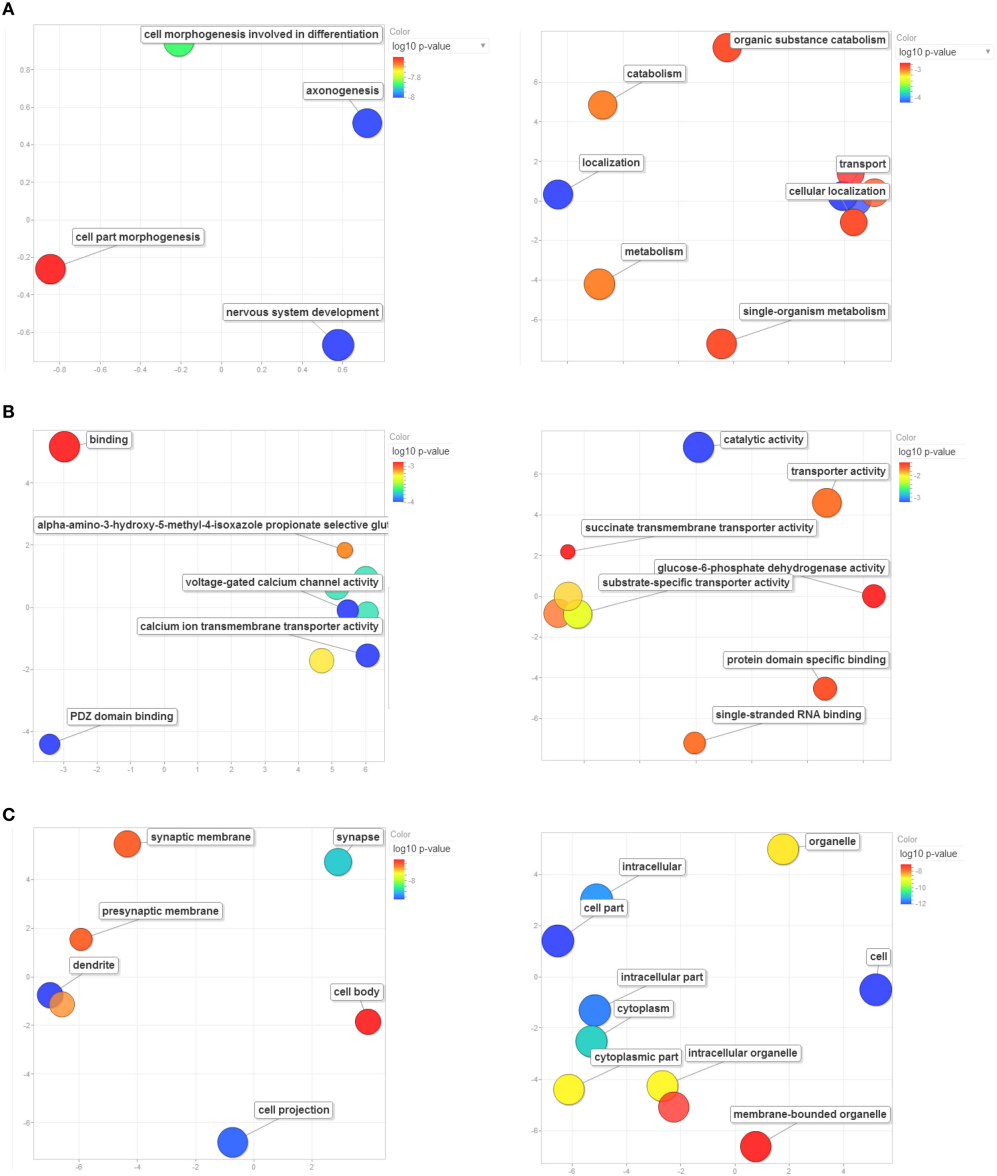
**Enrichment analysis based on GO terms for Master PC1's (left column) and Master PC2's (right column) top-500 correlated gene set**. Significant enrichment of genes included in GO terms relevant to **(A)** biological processes, **(B)** molecular functions, and **(C)** cellular components. Only significantly enriched GO categories are depicted. Bubbles are color-coded according to the adjusted log_10_ *p*-value of the top-correlated genes enrichment relative to the whole mouse genome. Blue and green bubbles are GO terms with more significant *p*-values than the orange and red bubbles. The bubbles' *x* and *y* coordinates were derived by applying multidimensional scaling to a matrix of the GO terms' semantic similarities; consequently, their closeness on the plot should closely reflect their closeness in the GO graph structure i.e. the semantic similarity. GO terms are based on the Gene Ontology Consortium available at: http://www.geneontology.org/. GO enrichment analysis was performed using WebGestalt: an integrated system for exploring gene sets in various biological contexts (Zhang et al., 2005), available at: http://bioinfo.vanderbilt.edu/webgestalt/.

For each of the Master PCs, the top-500 correlations across all records of the Amygdala Cohort Database, ranked by the Genetic Correlation (Spearman's rho), were strong (absolute value ranging from 0.7 to 0.9), and highly significant (*p* < 10^−8^, Supplementary Tables 5, 6). Enrichment analysis revealed 29 significantly enriched GO categories for each one of the top-500-correlated gene lists (Supplementary Tables 7, 8). Enrichment patterns relevant to biologic processes (Figure 7A), molecular function (Figure 7B), and cell compartment (Figure 7C) corresponding to each one of the genetic components were strikingly distinct. The first genetic component shared across all disorders seems to be involved in CNS development and located preferentially in neural projections (*p*_adj_ ~ 10^−8^ each). As such, it was specifically implicated in axonogenesis and dendrite formation (*p*_adj_ ~ 10^−8^ for both). This component was also enriched in genes located to the synapse (*p*_adj_ ~ 10^−7^), with special focus on calcium influx through both voltage-gated and ligand-gated (AMPA-receptor) channels (*p*_adj_ ~ 10^−4^ and *p*_adj_ ~ 10^−3^, respectively). On the other hand, the second genetic component shared across all disorders was mainly enriched in genes whose products are located in the cytoplasm (*p*_adj_ ~ 10^−11^) and are involved in catalyzing metabolic processes (*p*_adj_ ~ 10^−3^) as well as in facilitating localization and transport (*p*_adj_ ~ 10^−3^ each) of various substrates and in binding of both proteins and RNA (*p*_adj_ ~ 10^−2^ each).

## Discussion

In the present study we have systematically evaluated common and distinct gene sets and biological processes associated with major neuropsychiatric disorders using multiple, convergent approaches. A key problem that we had to address in this study was how to go about generating minimally biased lists of genes linked to each of the six highly diverse types of psychiatric disorders. We have benefitted greatly from large GWASs that systematically survey most of the genome. The GWAS coverage for the six disorders we have analyzed was far from uniform, in the case of SCZ the top-51 genes were extracted from a larger number of studies than ASD and most notably for anxiety disorders. These differences in coverage might introduce some bias in our list, which we have tried to avoid by analyzing similarly sized gene sets.

We have extracted a well-curated list of 180 genes based on top findings in GWAS with *p*-values of <10^−5^ across six psychiatric disorders: ADHD, Anx, ASD, BD, MDD, SCZ. A set of genes shows overlaps between five out of the six disorders (*n* = 15). Interestingly, genes with association to Anx show poor overlap (1/16 overlaps with ASD and 1/16 overlaps with MDD). Small overlap of Anx genes with other neuropsychiatric disorders might point to their distinct character but might also be explained by lower number of genes identified by GWAS with *p*-values of 10^−5^.

Two common approaches were used to investigate shared functions of genes associated with major neuropsychiatric disorders: protein-protein interactions and co-expression. Analysis of protein-protein interactions did not show any meaningful interconnected modules, which might be influenced by the fact that protein-protein interaction databases are still largely incomplete, and a number of unreported interactions may occur (Mosca et al., 2013). Next to direct protein-protein interactions, genetic interactions from model organisms, and interactions within pathways can be valuable information for a functional relation between seemingly unrelated genes. Spatiotemporal analysis of gene expression correlation in human brain (using BrainSpan developmental transcriptome data; Kang et al., 2011) has identified three co-expression modules. Although GO enrichment of the whole list (180 genes) did not highlight any functional categories, analysis of the co-expressed genes resulted in enrichment of the modules. This suggests that co-expression is a meaningful factor in exploring disease gene specificity. The biological process with highest enrichment of the largest module (*n* = 58 genes) was regulation of gene expression, the second largest module (*n* = 39 genes) was enriched for neuron projection guidance and the smallest module (*n* = 17 genes) showed enrichment in response to stress. It is of note that genes from Anx list were enriched in this small module.

Despite the fact that GO enrichments for the second and third module did not withstand correction for multiple testing, the enriched GO categories we identified represent molecular targets that have previously been implicated in different neuropsychiatric disorders and/or processes and cellular organelles for which involvement in neuropsychiatric disorders makes biological sense. For instance, regulation of gene expression for several genes, such as BDNF, has been previously implicated in various psychiatric disorders (Boulle et al., 2012). Moreover, differential expression of genes with a postulated role in the formation of neural projections, such as *AHI1*, have been implicated in psychiatric disorders such as SCZ (Amann-Zalcenstein et al., 2006; Ingason et al., 2010; Slonimsky et al., 2010), and ASD (Alvarez Retuerto et al., 2008) as well as in modulation of emotional phenotypes and stress vulnerability in relevant *Ahi1* knockout mouse models (Xu et al., 2010; Lotan et al., 2014). The fact that these same processes came out when using the current approach is intriguing. The approach we used, which is essentially hypothesis-free owing to its reliance on GWASs, differs from the approaches undertaken by the studies mentioned above, which focused a priori on relevant candidate genes. By doing so, the current findings arguably provide further rationale for targeting these processes in the context of neuropsychiatric disorders.

Next to traditional resources (represented by protein-protein interaction, weighted co-expression network, and GO), we took advantage of cross-species phenotypes data associated with major neuropsychiatric disorder genes (Kohler et al., 2013). We have identified specific phenotypes related to behavior, cognition, neuronal morphology, and neuronal activity to be over-represented in 110 genes associated with major neuropsychiatric disorders. Orthologous phenotypes, so called phenologs, have been shown to reveal functionally coherent gene networks, which can serve as models for systematic discovery of unique genes associated with disease (Mcgary et al., 2010); Woods et al. (2013) have shown predictions for atrial fibrillation, epilepsy and seizures using phenolog networks. Moreover, defining disease-specific phenotypes is initial step toward cross-species phenotype-expression correlation, which may add to translational validity of cross-species approaches.

We show that NHGRI-cross-disorder genes are enriched in the PSD, a dense and highly specialized structure in postsynaptic membrane of neurons. The proteins localizing to the PSD have been identified through proteomic studies (Bayes et al., 2011), where they function to concentrate and organize neurotransmitter receptors in the synaptic cleft and ensure for proper communication between neurons. Deficits in neuronal communication, as a consequence of impaired synaptic plasticity have been proposed as one of the common causes of neuropsychiatric disorders but significance of this observation has not been demonstrated. Results of the enrichment analysis of genes linked to six major neuropsychiatric disorders therefore confirm the role of PSD in disease pathology.

Looking into tissue specificity by using simplified information linking each gene to tissue with highest expression revealed that genes associated to SCZ, BD, and MDD are not highly expressed only in brain but also in other tissues, such as white blood. Association of immune system disturbances with SCZ has been already described (Miller et al., 2011) and current research has been to a greater extent focusing on interface between immunology and mental illness (Arolt et al., 2002).

Based on observed cohesivity in gene expression and enrichment in cross-species phenotypes, we focused on utilization of expression-phenotype correlation for analysis of genetic components shared across disorders. To achieve this, we took advantage of GeneNetwork—a web-based internet resource that embeds coherent behavior, genotype, and expression data from 28 genotypes of BXD mice (Wang et al., 2003). BXD mice were generated by crosses of C57BL/6J and DBA/2 inbred strains and all progenies are fully inbred strains with different parent haplotypes, which makes them an ideal model for study of correlations between RNA expression and biological traits and mapping the traits to QTLs.

When establishing the mouse orthologs of the NHGRI-cross-disorder gene sets, the amygdala region was chosen based upon biological and practical considerations. Biologically, it is a key region for regulation of emotion and has been consistently implicated in the pathogenesis of all major neuropsychiatric disorders (Catani et al., 2013). Moreover, structural and functional connectivity of the amygdala is involved in modulating complex phenotypes at the cognitive-emotional interface (Lotan et al., 2014), and is thus highly relevant across mental disorders. Practically, based upon estimates of mRNA expression for 50 genotypes of BXD mice in the basolateral region with balanced samples of males and females, the amygdala data set is one of the larger and higher quality “expression genetics” data sets available within the BXD database, with good annotation and high coverage by the array. In line with these observations, probes with at least moderate expression values could indeed be identified for the vast majority of relevant mouse orthologs. Theoretically, other regions such as prefrontal cortex, hippocampus, midbrain, and striatum could also have been profiled for establishing the mouse orthologs. Although comparing orthologs from different brain regions could provide complementary information, as different regions within the BXD database were profiled using different array platforms, direct comparison might be difficult.

In order to determine whether analysis of genetic components shared across disorders could be performed using the elegant GeneNetwork platform based on the BXD database, the feasibility of a cross-species approach needed to be demonstrated. Specifically, we initially wanted to assess the translational validity of such an analysis by establishing (expected) correlations between expression patterns of the mouse orthologs of the NHGRI-cross-disorder gene sets and relevant behavioral traits. To this end, we selected two different neuropsychiatric disorders, anxiety disorders and SCZ, and correlated their mouse-ortholog expression profile with relevant behavioral phenotype. As we aimed to use common paradigms, anxiety-related behavior was assessed with classic approach–avoidance anxiety tests (Cryan and Sweeney, 2011), while SCZ-related phenotypes were assessed with the prepulse inhibition paradigm that is indicative of disrupted sensorimotor gating (Powell et al., 2012). Notably, prepulse inhibition, formerly regarded as having construct and predictive validity for the psychotic domain of SCZ, has been recently considered to reflect a unique endophenotype that is at the interface of psychosis and cognition. As such, and going beyond SCZ, deficits in prepulse inhibition have also been implicated across a spectrum of affective disorders (Kohl et al., 2013). Moreover, it has recently been suggested that this paradigm could predict the impact of drugs or psychotherapy on cognitive performance in neuropsychiatric patients (Koch et al., 2014). Hence, our expression-phenotype results regarding anxiety and prepulse inhibition support the translational validity of the cross-species analytic approach that we have used in this manuscript.

In doing the cross-species analysis, there had been two relevant approaches for capturing the cross-disorder “essence” (if indeed there is such an essence). In the first approach, a PCA limited only to genes that are shared by most disorders would have been performed. We thought this option had two major disadvantages: (a) No single gene was associated with all six disorders, making the cross-disorder analysis *a priori* somewhat incomplete, and (b) from the clinical perspective, as the boundaries between disorders (i.e., the phenotype) are often vague and arbitrary, focusing only on genes that are shared by a minimal number of (supposedly) distinct disorders may be inherently subject to the same flaws of the current diagnostic system (Faravelli et al., 2012). In the second approach, one starts with the entire NHGRI-cross-disorder gene set, which obviously includes many genes that are not shared across disorders, and then uses a two-stage PCA. In this sort of analysis, the mouse neurobiology (or BXD database) identifies the cross-disorder genetic components. Obviating the need of relying on previous assumptions regarding the validity of current psychiatric classification, such data-driven approaches could offer more valid identification of independent components in neurobiological systems (Wessel and Ullsperger, 2011). In the current manuscript, we have used a combination of both approaches; while the first was implemented in the human-based analysis, the second approach was used for the cross-species analysis. Naturally, in the future other possibilities for analyzing the data could be implemented.

The data suggesting the existence of significant cross-disorder genetic components sheds important light on the ongoing debate concerning the current classification systems in psychiatry. Current diagnostic systems for mental disorders rely upon presenting signs and symptoms, with the result that current definitions do not adequately reflect relevant neurobiological and behavioral systems—impeding not only research on etiology and pathophysiology, but also the development of new treatments (Cuthbert and Insel, 2013). The NIMH began the RDoC project in 2009 to develop a research classification system for mental disorders based upon dimensions of neurobiology and observable behavior. RDoC supports research to explicate fundamental dimensions that cut across current heterogeneous disorder categories, realizing the fact that future diagnostic systems will largely depend on ongoing advances in genetics and neuroscience. In this respect, the robust cross-diagnostic data presented above, pointing to highly shared, albeit modest, genetic components that lies at the core of the major neuropsychiatric disorders, yields support to the dimensional approach outlined.

After verifying that both Master PCs receive substantial contribution from each and every disorder, we tried to elucidate their biological meaning. A main computational advantage of the GeneNetwork platform is that it treats synthetic PCs as independent traits, thus enabling the user to correlate them with other traits of interest. Thus, for the enrichment analysis, we chose to include the top-500 genes that co-express with each Master PC across the BXD strains' amygdalae. While the relatively large gene list enables adequate power to detect enrichment relative to the reference set, the high correlation coefficients between all 500 genes and their respective Master PC ensures that the genes used for the enrichment analysis do in fact closely resemble the vector of the Master PC that they are intended to reflect.

Results of the GO-based enrichment analysis suggested that the two genetic components shared across all disorders represent distinct sets of biological processes, molecular functions and cellular components. The first genetic component seems to be involved in CNS development, located preferentially in neural projections and synapse, implicated in axonogenesis and dendrite formation and modulates calcium influx through glutamatergic neurotransmission. As all of these processes and functions have been extensively implicated across all neuropsychiatric disorders, the contribution of such a genetic component to all major disorders seems highly plausible. Moreover, many of these molecular functions, such as calcium influx and excitatory neurotransmission, are targeted by the currently available psychoactive and other somatic treatments (Baldinger et al., 2014), thus granting this genetic component predictive validity as well. Notably, the “neural projections” GO term was also significantly enriched in our enrichment analysis of cross-disorder genes co-expressed during human brain development (blue module, Figure 5). The fact that two different analytical approaches converged on the same biological process lends further support to its common and central role in the pathogenesis of common neuropsychiatric disorders.

In contrast to the first genetic component, the second genetic component shared across all disorders was mainly enriched in genes whose products are located in the cytoplasm and are involved in catalyzing metabolic processes as well as in facilitating localization and transport of various substrates and in binding of both proteins and RNA. Although these intra-cellular processes do not “pop-up” as the “usual suspect” when it comes to neuropsychiatric disorders, they have been consistently implicated in neuropsychiatric disorders such as SCZ (Prabakaran et al., 2004), BD (Baek et al., 2013), ASD (Raymond et al., 2014), and MDD (Hoyo-Becerra et al., 2014). Moreover, many psychotropic drugs seem to modulate such intra-cellular processes, adding further predictive validity to this genetic component (Lauterbach, 2013). Importantly, as mentioned above with respect to “neural projections,” protein binding and regulation of metabolic processes pop-out also in our GO enrichment results from cross-disorder genes co-expressed in developing human brain (turquoise module, Figure 4), highlighting once again the convergence of different analytical approaches on similar biological processes.

In addition to the genetic components shared across disorders, the data suggest that the majority of genes and factor loading associated with each specific disorder is unique. In this respect, it could be hypothesized that a common (pathologic) molecular infrastructure located to neural projections, cytoplasm (or possibly both) may be necessary to induce a primary vulnerability to develop a neuropsychiatric disorder. Further distinct molecular processes which build-up on top of this common infrastructure ultimately lead, in certain patients, to the development of one or another specific neuropsychiatric disorder.

One limitation of the current study is that although logical and plausible, the above-mentioned hypothesis is somewhat speculative as it cannot be induced straightforwardly from our results. For instance, there is no way to know from the current GWASs design which combinations of genes are associated with a clinical disorder at the individual patient level. Moreover, GWAS derives data points to genes, not to causative mutations, which may alter gene expression, protein structure or both. Needless to say that addressing this complexity is beyond the scope and resolution of the analysis presented in this manuscript. Another important limitation of the current study is its reliance, on part, on data obtained from animal models of neuropsychiatric disorders. Many of the symptoms of major neuropsychiatric disorders are dependent on the processing of complex psychological and cognitive concepts that clearly cannot be measured in animals, such as paranoid delusions or “fear of losing control or going crazy.” It is thus clear from the clinical presentation of these disorders that they can never be fully emulated as a syndrome in animals (Crawley, 2007). Therefore, our findings derived from the cross-species approach are inherently limited in their generalizability upon translation back to humans. Nevertheless, given the substantial conservation of genetic, neurochemical and neuroanatomical features seen across mammals (Arguello and Gogos, 2006), theoretically, studying the genetic determinants of animal behavioral response, could, by inference, promote our understanding of the genetic basis of human behavior under both normal and pathological states (Cryan and Holmes, 2005).

## Concluding remarks

We have combined genetic risk factors identified in SCZ, BD, ADHD, ASD, MDD, and Anx disorders based on top findings in GWAS from NHGRI catalog. We have scored these genes based on the highest number of overlap between these disorders and found 15 genes affected in five disorders, 20 affected in four or more disorders, 28 genes affected in three or more disorders, and 39 genes with overlap between two disorders. 141 genes do not overlap.

We demonstrated that although these disorders share a relatively small set of genes, there are two fundamental yet distinct genetic components, or vectors, that are both shared by all six disorders. While the **first** component is involved in CNS development, neural projections and synaptic transmission, the **second** component is implicated in various cytoplasmic organelles and cellular processes such as metabolism, transport and binding. Although both components were implicated in each and every disorder, their overall genetic (and possibly pathophysiologic) contribution to the development of common neuropsychiatric disorders may be modest.

## Author contributions

Amit Lotan performed analysis of BXD mouse data: PCA on amygdala gene expression, expression phenotype correlation and identification of distinct genetic components with Master traits, and wrote the manuscript. Michaela Fenckova performed protein-protein interaction network analysis, enrichment and co-expression analysis and wrote the manuscript. Monique van der Voet curated gene sets, contributed to manuscript concept, and wrote the manuscript. Janita Bralten contributed to manuscript concept and to writing of the manuscript. Aet Alttoa and Luanna Dixson contributed to manuscript concept and performed analyses that were not used in the final version of the manuscript. Robert W. Williams contributed to manuscript concept and reviewed the manuscript.

### Conflict of interest statement

The authors declare that the research was conducted in the absence of any commercial or financial relationships that could be construed as a potential conflict of interest.

## References

[B1] Alvarez Retuerto A. I. Cantor R. M. Gleeson J. G. Ustaszewska A. Schackwitz W. S. Pennacchio L. A.. (2008). Association of common variants in the Joubert syndrome gene (AHI1) with autism. Hum. Mol. Genet. 17, 3887–3896. 10.1093/hmg/ddn291 18782849 PMC2638573

[B2] Amann-Zalcenstein D. Avidan N. Kanyas K. Ebstein R. P. Kohn Y. Hamdan A.. (2006). AHI1, a pivotal neurodevelopmental gene, and C6orf217 are associated with susceptibility to schizophrenia. Eur. J. Hum. Genet. 14, 1111–1119. 10.1038/sj.ejhg.5201675 16773125

[B3] Arguello P. A. Gogos J. A. (2006). Modeling madness in mice: one piece at a time. Neuron 52, 179–196. 10.1016/j.neuron.2006.09.023 17015235

[B4] Arolt V. Rothermundt M. Peters M. Leonard B. (2002). Immunological research in clinical psychiatry: report on the consensus debate during the 7th Expert Meeting on Psychiatry and Immunology. Mol. Psychiatry 7, 822–826. 10.1038/sj.mp.4001115 12232771

[B5] Baek J. H. Bernstein E. E. Nierenberg A. A. (2013). One-carbon metabolism and bipolar disorder. Aust. N. Z J. Psychiatry 47, 1013–1018. 10.1177/0004867413502091 23969624

[B6] Baldinger P. Lotan A. Frey R. Kasper S. Lerer B. Lanzenberger R. (2014). Neurotransmitters and electroconvulsive therapy. J. ECT 30, 116–121. 10.1097/YCT.0000000000000138 24820941

[B7] Bayes A. van De Lagemaat L. N. Collins M. O. Croning M. D. Whittle I. R. Choudhary J. S.. (2011). Characterization of the proteome, diseases and evolution of the human postsynaptic density. Nat. Neurosci. 14, 19–21. 10.1038/nn.2719 21170055 PMC3040565

[B8] Benes F. M. (2010). Amygdalocortical circuitry in schizophrenia: from circuits to molecules. Neuropsychopharmacology 35, 239–257. 10.1038/npp.2009.116 19727065 PMC3055447

[B9] Benjamini Y. Hochberg Y. (1995). Controlling the false discovery rate: a practical and powerful approach to multiple testing. J. Roy. Statist. Soc. Ser. B (Methodol.) 57, 289–300 10.2307/2346101

[B10] Boulle F. Van Den Hove D. L. Jakob S. B. Rutten B. P. Hamon M. van Os J.. (2012). Epigenetic regulation of the BDNF gene: implications for psychiatric disorders. Mol. Psychiatry 17, 584–596. 10.1038/mp.2011.107 21894152

[B11] Braff D. L. Geyer M. A. Swerdlow N. R. (2001). Human studies of prepulse inhibition of startle: normal subjects, patient groups, and pharmacological studies. Psychopharmacology (Berl.) 156, 234–258. 10.1007/s002130100810 11549226

[B12] Catani M. Dell'acqua F. Thiebaut De Schotten M. (2013). A revised limbic system model for memory, emotion and behaviour. Neurosci. Biobehav. Rev. 37, 1724–1737. 10.1016/j.neubiorev.2013.07.001 23850593

[B13] Crawley J. N. (2007). What's Wrong With my Mouse?: Behavioral Phenotyping of Transgenic and Knockout Mice. Hoboken, NJ: Wiley; Chichester: John Wiley.

[B70] Cross-Disorder Group of the Psychiatric Genomics Consortium. (2013). Identification of risk loci with shared effects on five major psychiatric disorders: a genome-wide analysis. Lancet 381, 1371–1379. 10.1016/S0140-6736(12)62129-1 23453885 PMC3714010

[B15] Cryan J. F. Holmes A. (2005). The ascent of mouse: advances in modelling human depression and anxiety. Nat. Rev. Drug Discov. 4, 775–790. 10.1038/nrd1825 16138108

[B16] Cryan J. F. Sweeney F. F. (2011). The age of anxiety: role of animal models of anxiolytic action in drug discovery. Br. J. Pharmacol. 164, 1129–1161. 10.1111/j.1476-5381.2011.01362.x 21545412 PMC3229755

[B17] Cuthbert B. N. Insel T. R. (2013). Toward the future of psychiatric diagnosis: the seven pillars of RDoC. BMC Med. 11:126. 10.1186/1741-7015-11-126 23672542 PMC3653747

[B18] Dammer E. B. Duong D. M. Diner I. Gearing M. Feng Y. Lah J. J.. (2013). Neuron enriched nuclear proteome isolated from human brain. J. Proteome Res. 12, 3193–3206. 10.1021/pr400246t 23768213 PMC3734798

[B19] Donovan L. E. Higginbotham L. Dammer E. B. Gearing M. Rees H. D. Xia Q.. (2012). Analysis of a membrane-enriched proteome from postmortem human brain tissue in Alzheimer's disease. Proteomics Clin. Appl. 6, 201–211. 10.1002/prca.201100068 22532456 PMC3338199

[B20] Dziobek I. Bahnemann M. Convit A. Heekeren H. R. (2010). The role of the fusiform-amygdala system in the pathophysiology of autism. Arch. Gen. Psychiatry 67, 397–405. 10.1001/archgenpsychiatry.2010.31 20368515

[B69] Eden E. Navon R. Steinfeld I. Lipson D. Yakhini Z. (2009). GOrilla: a tool for discovery and visualization of enriched go terms in ranked gene lists. BMC Bioinformatics 10:48. 10.1186/1471-2105-10-48 19192299 PMC2644678

[B21] Faraone S. V. Biederman J. Wozniak J. (2012). Examining the comorbidity between attention deficit hyperactivity disorder and bipolar I disorder: a meta-analysis of family genetic studies. Am. J. Psychiatry 169, 1256–1266. 10.1176/appi.ajp.2012.12010087 23212057

[B22] Faravelli C. Castellini G. Landi M. Brugnera A. (2012). Are psychiatric diagnoses an obstacle for research and practice? Reliability, validity and the problem of psychiatric diagnoses. The case of GAD. Clin. Pract. Epidemiol. Ment. Health 8, 12–15. 10.2174/1745017901208010012 22408646 PMC3293164

[B23] Foussias G. Agid O. Fervaha G. Remington G. (2014). Negative symptoms of schizophrenia: clinical features, relevance to real world functioning and specificity versus other CNS disorders. Eur. Neuropsychopharmacol. 24, 693–709. 10.1016/j.euroneuro.2013.10.017 24275699

[B24] Hamilton J. P. Chen M. C. Waugh C. E. Joormann J. Gotlib I. H. (2014). Distinctive and common neural underpinnings of major depression, social anxiety, and their comorbidity. Soc. Cogn. Affect. Neurosci. [Epub ahead of print]. 10.1093/scan/nsu084 25038225 PMC4381237

[B25] Hettema J. M. Neale M. C. Kendler K. S. (2001). A review and meta-analysis of the genetic epidemiology of anxiety disorders. Am. J. Psychiatry 158, 1568–1578. 10.1176/appi.ajp.158.10.1568 11578982

[B71] Hindorff L. A. MacArthur J. Morales J. Junkins H. A. Hall P. N. Klemm A. K. (2014). A catalog of published genome-wide association studies. Available online at: www.genome.gov/gwastudies. (Accessed June 14, 2014).

[B26] Hoyo-Becerra C. Schlaak J. F. Hermann D. M. (2014). Insights from interferon-alpha-related depression for the pathogenesis of depression associated with inflammation. Brain Behav. Immun. [Epub ahead of print]. 10.1016/j.bbi.2014.06.200 25066466

[B27] Huang Da W. Sherman B. T. Lempicki R. A. (2009a). Bioinformatics enrichment tools: paths toward the comprehensive functional analysis of large gene lists. Nucleic Acids Res. 37, 1–13. 10.1093/nar/gkn923 19033363 PMC2615629

[B28] Huang Da W. Sherman B. T. Lempicki R. A. (2009b). Systematic and integrative analysis of large gene lists using DAVID bioinformatics resources. Nat. Protoc. 4, 44–57. 10.1038/nprot.2008.211 19131956

[B29] Ingason A. Giegling I. Cichon S. Hansen T. Rasmussen H. B. Nielsen J.. (2010). A large replication study and meta-analysis in European samples provides further support for association of AHI1 markers with schizophrenia. Hum. Mol. Genet. 19, 1379–1386. 10.1093/hmg/ddq009 20071346 PMC2838541

[B30] Insel T. R. (2014). The NIMH Research Domain Criteria (RDoC) Project: precision medicine for psychiatry. Am. J. Psychiatry 171, 395–397. 10.1176/appi.ajp.2014.14020138 24687194

[B31] Joshi G. Faraone S. V. Wozniak J. Tarko L. Fried R. Galdo M.. (2014). Symptom profile of ADHD in youth with high-functioning autism spectrum disorder: a comparative study in psychiatrically referred populations. J. Atten. Disord. [Epub ahead of print]. 10.1177/1087054714543368 25085653 PMC4312732

[B32] Kang H. J. Kawasawa Y. I. Cheng F. Zhu Y. Xu X. Li M.. (2011). Spatio-temporal transcriptome of the human brain. Nature 478, 483–489. 10.1038/nature10523 22031440 PMC3566780

[B33] Kirov G. Pocklington A. J. Holmans P. Ivanov D. Ikeda M. Ruderfer D.. (2012). De novo CNV analysis implicates specific abnormalities of postsynaptic signalling complexes in the pathogenesis of schizophrenia. Mol. Psychiatry 17, 142–153. 10.1038/mp.2011.154 22083728 PMC3603134

[B34] Koch S. B. van Zuiden M. Nawijn L. Frijling J. L. Veltman D. J. Olff M. (2014). Intranasal oxytocin as strategy for medication-enhanced psychotherapy of PTSD: salience processing and fear inhibition processes. Psychoneuroendocrinology 40, 242–256. 10.1016/j.psyneuen.2013.11.018 24485496

[B35] Kohl S. Heekeren K. Klosterkotter J. Kuhn J. (2013). Prepulse inhibition in psychiatric disorders–apart from schizophrenia. J. Psychiatr. Res. 47, 445–452. 10.1016/j.jpsychires.2012.11.018 23287742

[B36] Kohler S. Doelken S. C. Ruef B. J. Bauer S. Washington N. Westerfield M.. (2013). Construction and accessibility of a cross-species phenotype ontology along with gene annotations for biomedical research. F1000Res. 2:30. 10.12688/f1000research.2-30.v2 24358873 PMC3799545

[B37] Lage K. (2014). Protein-protein interactions and genetic diseases: the interactome. Biochim. Biophys. Acta 1842, 1971–1980. 10.1016/j.bbadis.2014.05.028 24892209 PMC4165798

[B38] Langfelder P. Horvath S. (2008). WGCNA: an R package for weighted correlation network analysis. BMC Bioinformatics 9:559. 10.1186/1471-2105-9-559 19114008 PMC2631488

[B39] Lauterbach E. C. (2013). Neuroprotective effects of psychotropic drugs in Huntington's disease. Int. J. Mol. Sci. 14, 22558–22603. 10.3390/ijms141122558 24248060 PMC3856079

[B40] Loos M. Staal J. Pattij T. Neuro B. M. P. C. Smit A. B. Spijker S. (2012). Independent genetic loci for sensorimotor gating and attentional performance in BXD recombinant inbred strains. Genes Brain Behav. 11, 147–156. 10.1111/j.1601-183X.2011.00754.x 22098762

[B41] Lotan A. Lifschytz T. Slonimsky A. Broner E. C. Greenbaum L. Abedat S.. (2014). Neural mechanisms underlying stress resilience in Ahi1 knockout mice: relevance to neuropsychiatric disorders. Mol. Psychiatry 19, 243–252. 10.1038/mp.2013.123 24042478

[B42] Mcgary K. L. Park T. J. Woods J. O. Cha H. J. Wallingford J. B. Marcotte E. M. (2010). Systematic discovery of nonobvious human disease models through orthologous phenotypes. Proc. Natl. Acad. Sci. U.S.A. 107, 6544–6549. 10.1073/pnas.0910200107 20308572 PMC2851946

[B43] Mcteague L. M. Lang P. J. (2012). The anxiety spectrum and the reflex physiology of defense: from circumscribed fear to broad distress. Depress. Anxiety 29, 264–281. 10.1002/da.21891 22511362 PMC3612961

[B44] Miller B. J. Buckley P. Seabolt W. Mellor A. Kirkpatrick B. (2011). Meta-analysis of cytokine alterations in schizophrenia: clinical status and antipsychotic effects. Biol. Psychiatry 70, 663–671. 10.1016/j.biopsych.2011.04.013 21641581 PMC4071300

[B45] Morciano M. Burre J. Corvey C. Karas M. Zimmermann H. Volknandt W. (2005). Immunoisolation of two synaptic vesicle pools from synaptosomes: a proteomics analysis. J. Neurochem. 95, 1732–1745. 10.1111/j.1471-4159.2005.03506.x 16269012

[B46] Mosca R. Pons T. Ceol A. Valencia A. Aloy P. (2013). Towards a detailed atlas of protein-protein interactions. Curr. Opin. Struct. Biol. 23, 929–940. 10.1016/j.sbi.2013.07.005 23896349

[B47] Powell S. B. Weber M. Geyer M. A. (2012). Genetic models of sensorimotor gating: relevance to neuropsychiatric disorders. Curr. Top. Behav. Neurosci. 12, 251–318. 10.1007/7854_2011_195 22367921 PMC3357439

[B48] Prabakaran S. Swatton J. E. Ryan M. M. Huffaker S. J. Huang J.-J. Griffin J. L.. (2004). Mitochondrial dysfunction in schizophrenia: evidence for compromised brain metabolism and oxidative stress. Mol. Psychiatry 9, 684–697. 10.1038/sj.mp.4001532 15098003

[B49] Price J. L. Drevets W. C. (2010). Neurocircuitry of mood disorders. Neuropsychopharmacology 35, 192–216. 10.1038/npp.2009.104 19693001 PMC3055427

[B50] Rauch S. L. Shin L. M. Wright C. I. (2003). Neuroimaging studies of amygdala function in anxiety disorders. Ann. N. Y Acad. Sci. 985, 389–410. 10.1111/j.1749-6632.2003.tb07096.x 12724173

[B51] Raymond L. J. Deth R. C. Ralston N. V. (2014). Potential role of selenoenzymes and antioxidant metabolism in relation to autism etiology and pathology. Autism Res. Treat. 2014, 164938. 10.1155/2014/164938 24734177 PMC3966422

[B52] Segal E. Wang H. Koller D. (2003). Discovering molecular pathways from protein interaction and gene expression data. Bioinformatics 19(Suppl. 1), i264–i271. 10.1093/bioinformatics/btg1037 12855469

[B53] Slonimsky A. Levy I. Kohn Y. Rigbi A. Ben-Asher E. Lancet D.. (2010). Lymphoblast and brain expression of AHI1 and the novel primate-specific gene, C6orf217, in schizophrenia and bipolar disorder. Schizophr. Res. 120, 159–166. 10.1016/j.schres.2010.03.041 20452750

[B54] Spiegelhalder K. Regen W. Nanovska S. Baglioni C. Riemann D. (2013). Comorbid sleep disorders in neuropsychiatric disorders across the life cycle. Curr. Psychiatry Rep. 15, 364. 10.1007/s11920-013-0364-5 23636987

[B55] Spielberg J. M. Miller G. A. Warren S. L. Sutton B. P. Banich M. Heller W. (2014). Transdiagnostic dimensions of anxiety and depression moderate motivation-related brain networks during goal maintenance. Depress. Anxiety. [Epub ahead of print]. 10.1002/da.22271 24753242 PMC4418555

[B56] Stauch K. L. Purnell P. R. Fox H. S. (2014). Quantitative proteomics of synaptic and nonsynaptic mitochondria: insights for synaptic mitochondrial vulnerability. J. Proteome Res. 13, 2620–2636. 10.1021/pr500295n 24708184 PMC4015687

[B57] Stein M. B. Jang K. L. Livesley W. J. (1999). Heritability of anxiety sensitivity: a twin study. Am. J. Psychiatry 156, 246–251. 9989561 10.1176/ajp.156.2.246

[B58] Supek F. Bosnjak M. Skunca N. Smuc T. (2011). REVIGO summarizes and visualizes long lists of gene ontology terms. PLoS ONE 6:e21800. 10.1371/journal.pone.0021800 21789182 PMC3138752

[B59] van Den Buuse M. (2010). Modeling the positive symptoms of schizophrenia in genetically modified mice: pharmacology and methodology aspects. Schizophr. Bull. 36, 246–270. 10.1093/schbul/sbp132 19900963 PMC2833124

[B60] Vilella A. J. Severin J. Ureta-Vidal A. Heng L. Durbin R. Birney E. (2009). EnsemblCompara genetrees: complete, duplication-aware phylogenetic trees in vertebrates. Genome Res. 19, 327–335. 10.1101/gr.073585.107 19029536 PMC2652215

[B61] Wang J. Duncan D. Shi Z. Zhang B. (2013). WEB-based GEne SeT AnaLysis Toolkit (WebGestalt): update 2013. Nucleic Acids Res. 41, W77–W83. 10.1093/nar/gkt439 23703215 PMC3692109

[B62] Wang J. Williams R. W. Manly K. F. (2003). WebQTL: web-based complex trait analysis. Neuroinformatics 1, 299–308. 10.1385/NI:1:4:299 15043217

[B63] Welter D. Macarthur J. Morales J. Burdett T. Hall P. Junkins H.. (2014). The NHGRI GWAS Catalog, a curated resource of SNP-trait associations. Nucleic Acids Res. 42, D1001–D1006. 10.1093/nar/gkt1229 24316577 PMC3965119

[B64] Wessel J. R. Ullsperger M. (2011). Selection of independent components representing event-related brain potentials: a data-driven approach for greater objectivity. Neuroimage 54, 2105–2115. 10.1016/j.neuroimage.2010.10.033 20965258

[B65] Woods J. O. Singh-Blom U. M. Laurent J. M. Mcgary K. L. Marcotte E. M. (2013). Prediction of gene-phenotype associations in humans, mice, and plants using phenologs. BMC Bioinformatics 14:203. 10.1186/1471-2105-14-203 23800157 PMC3704650

[B66] Xu X. Yang H. Lin Y. F. Li X. Cape A. Ressler K. J.. (2010). Neuronal Abelson helper integration site-1 (Ahi1) deficiency in mice alters TrkB signaling with a depressive phenotype. Proc. Natl. Acad. Sci. U.S.A. 107, 19126–19131. 10.1073/pnas.1013032107 20956301 PMC2973903

[B67] Yang R. J. Mozhui K. Karlsson R. M. Cameron H. A. Williams R. W. Holmes A. (2008). Variation in mouse basolateral amygdala volume is associated with differences in stress reactivity and fear learning. Neuropsychopharmacology 33, 2595–2604. 10.1038/sj.npp.1301665 18185497

[B68] Zhang B. Kirov S. Snoddy J. (2005). WebGestalt: an integrated system for exploring gene sets in various biological contexts. Nucleic Acids Res. 33, W741–W748. 10.1093/nar/gki475 15980575 PMC1160236

