# Supplementary material for: Neuroinformatic analyses of common and distinct genetic components associated with major neuropsychiatric disorders
Source: Front Neurosci. 2014 Nov 6;8:331. doi: 10.3389/fnins.2014.00331 (PMC4222236; doi:10.3389/fnins.2014.00331)
Supplement: Supplementary file 1 [file DataSheet1.ZIP › Supplementary Material/Suppl_material_legends.docx]

**SUPPLEMENTARY MATERIAL**

**TABLE 1 | Genes associated to major neuropsychiatric disorders. (A)** Publications describing GWAS for major psychiatric disorders. **(B)** Top-51 protein coding genes for each psychiatric disorder, retrieved from NHGRI SNPs with a *p*-value < 1.0 x 10^-5^.

**TABLE 2 | Frequency of cross-species phenotypes in NHGRI-cross-disorder genes mapped from Uberpheno.**

**TABLE 3 | Genes** **in all co-expressed modules and their connectivity.**

**TABLE 4 | Disease gene enrichment in co-expressed modules.**

**TABLES 5 and 8** **|** Top-500 correlations across all records of the Amygdala Cohort Database, ranked by the Genetic Correlation (Spearman's rho) for Master PC 1 and 2, respectively.

**TABLES 6 and 7 |** Significantly enriched GO categories within the top-500-correlated gene lists for Master PC 1 and 2, respectively.

**TABLE 9 |** Probes for the top 16 protein-coding genes for anxiety disorders obtained from NHGRI identified within the INIA Amygdala Cohort Affy MoGene 1.0 ST (Mar11) RMA Database.

**TABLE 10 |** Probes for 46 of the top 51 protein-coding genes for SCZ obtained from NHGRI identified within the INIA Amygdala Cohort Affy MoGene 1.0 ST (Mar11) RMA Database.

**TABLE 11 |** Eight relevant anxiety-related traits, based upon [**Yang et al. 2008**](#_ENREF_44), identified in the BXD database.

**TABLE 12 |** Prepulse inhibition-related traits, based upon Loos et al [**Loos et al. 2012**](#_ENREF_27), identified in the BXD database.

**SUPPLEMENTARY FIGURE 1 | Direct protein-protein interactions in NHGRI-cross-disorder set.**

**SUPPLEMENTARY FIGURE 2 | Protein-protein interaction network of genes associated with five neuropsychiatric disorders including interactors connecting at least two proteins.** Non-disease interactors connecting NHGRI-cross-disorder proteins are depicted in white.

**SUPPLEMENTARY FIGURE 3** | Top synthetic expression PCs derived from the anxiety-related probe set (Supplementary Table 9).

**SUPPLEMENTARY FIGURE 4 |** Top synthetic expression PCs derived from the SCZ-related probe set (Supplementary Table 10).

**SUPPLEMENTARY FIGURE 5 |** Top two PCs derived from anxiety traits (Supplementary Table 11).

**SUPPLEMENTARY FIGURE 6 |** Top PC derived from prepulse inhibition traits (Supplementary Table 12).
