# Supplementary figures and images for: Neuroinformatic analyses of common and distinct genetic components associated with major neuropsychiatric disorders
Source: Front Neurosci. 2014 Nov 6;8:331. doi: 10.3389/fnins.2014.00331 (PMC4222236; doi:10.3389/fnins.2014.00331)

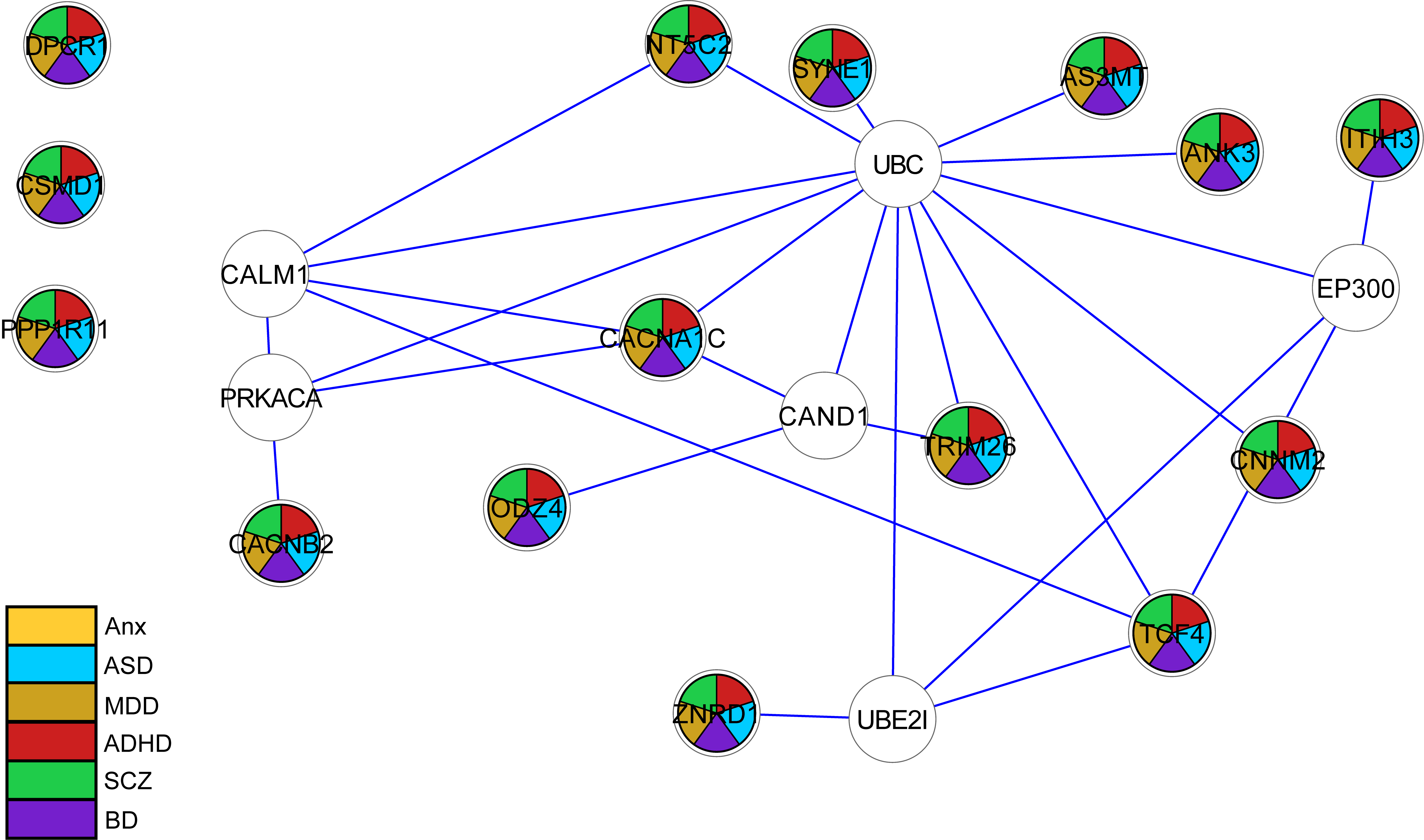

Supplement: Supplementary file 1 [file DataSheet1.ZIP › Supplementary Material/112662_Fenckova_Image_2.TIF]

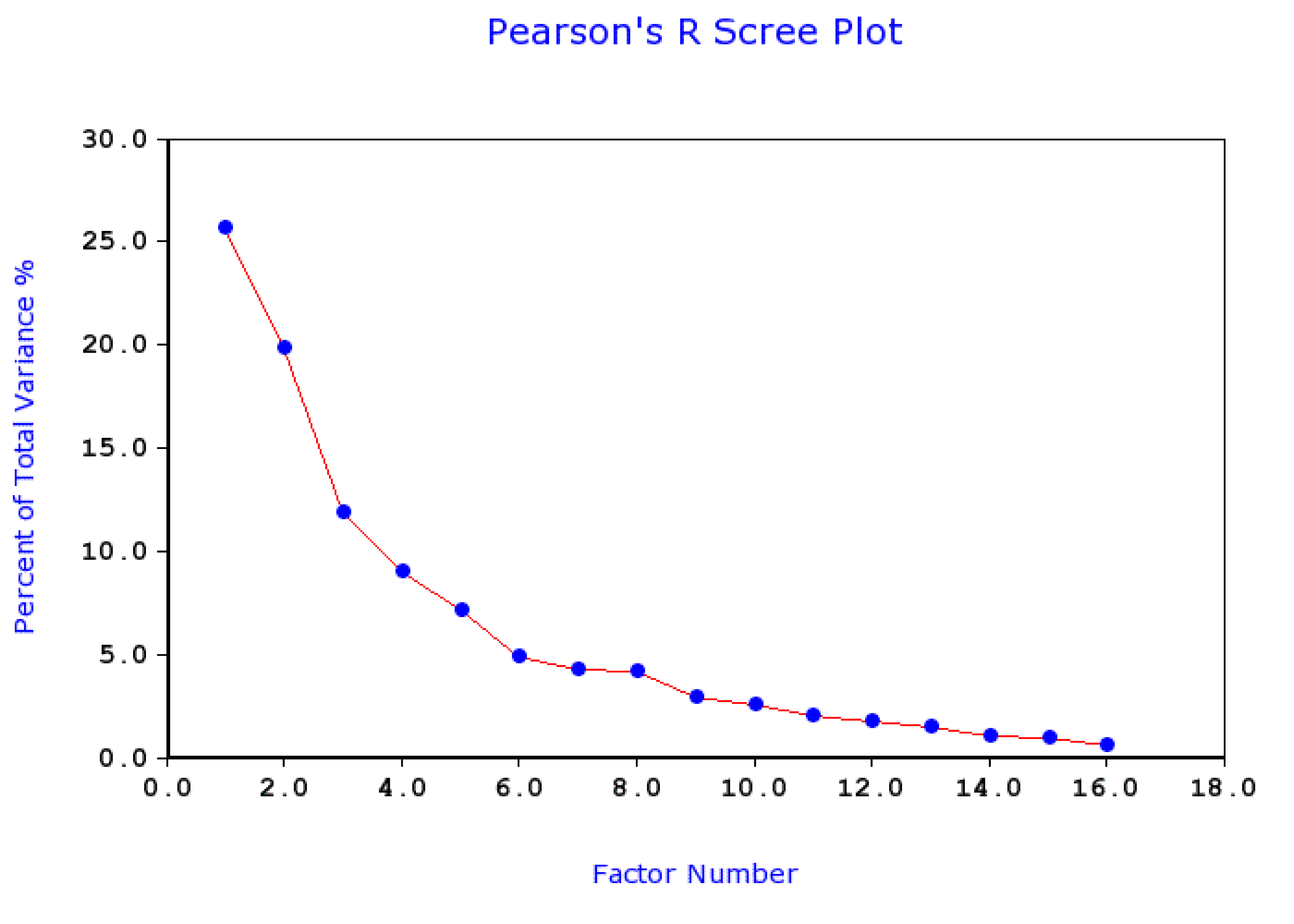

Supplement: Supplementary file 1 [file DataSheet1.ZIP › Supplementary Material/112662_Fenckova_Image_3.TIF]

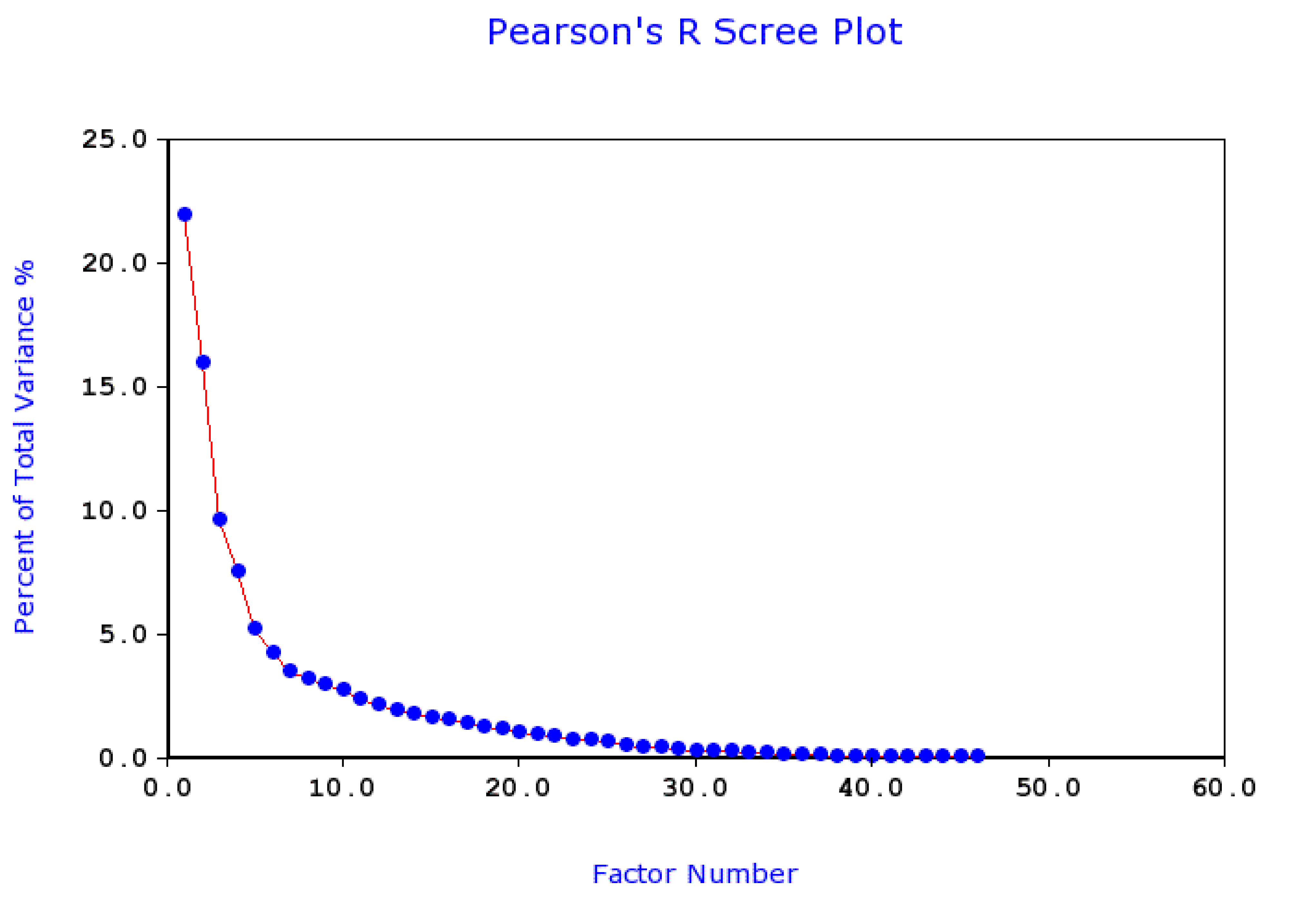

Supplement: Supplementary file 1 [file DataSheet1.ZIP › Supplementary Material/112662_Fenckova_Image_4.TIF]

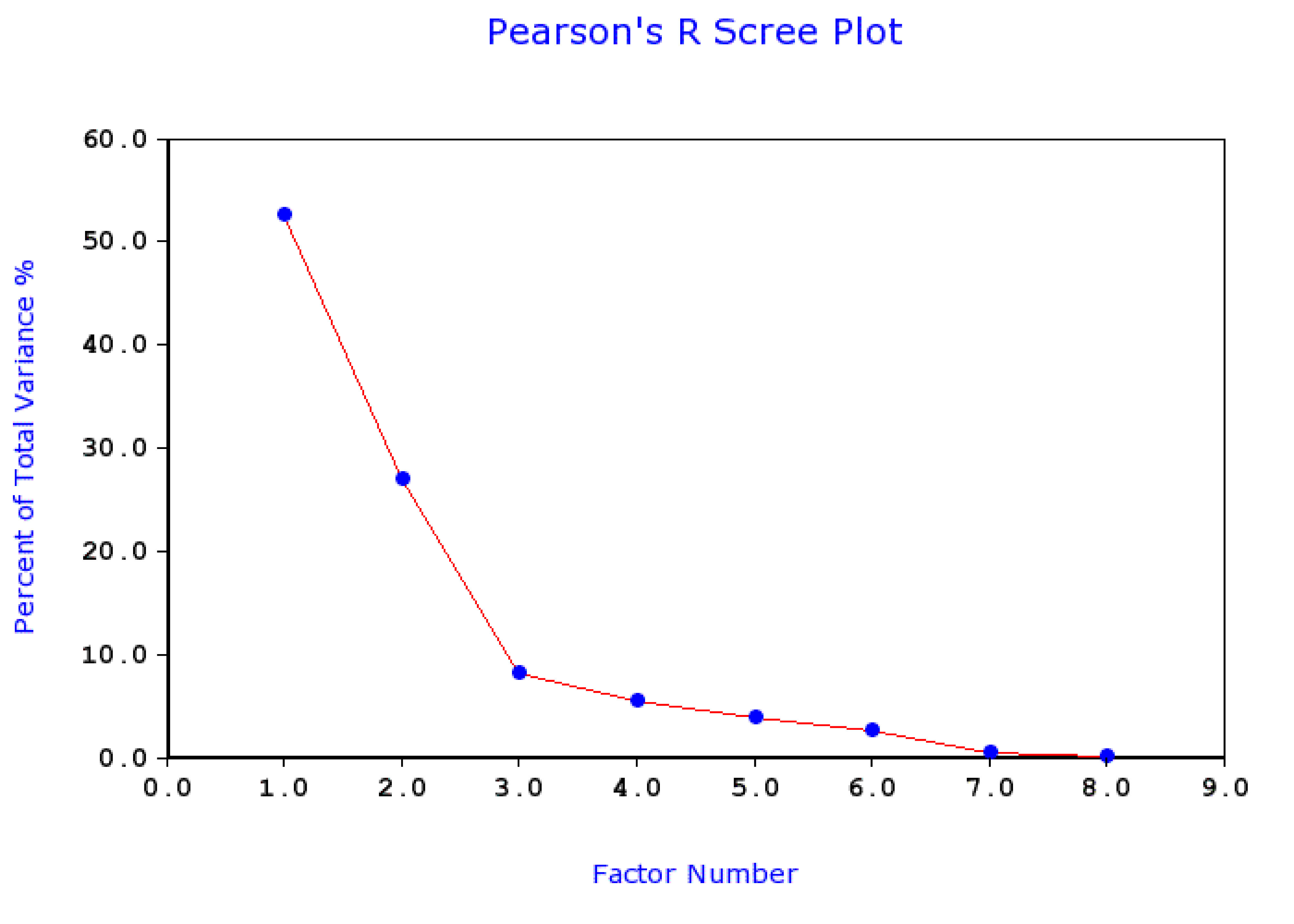

Supplement: Supplementary file 1 [file DataSheet1.ZIP › Supplementary Material/112662_Fenckova_Image_5.TIF]

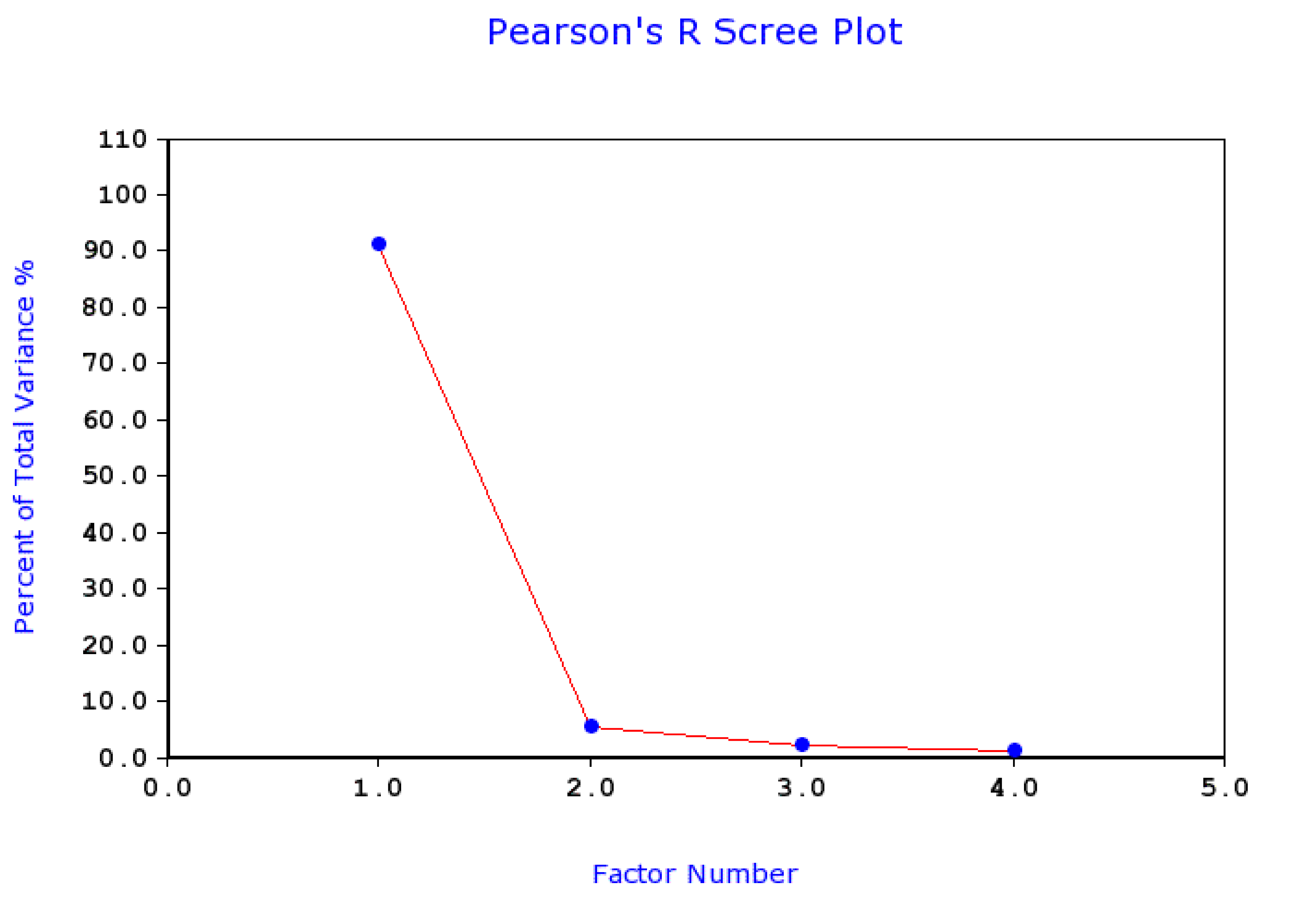

Supplement: Supplementary file 1 [file DataSheet1.ZIP › Supplementary Material/112662_Fenckova_Image_6.TIF]
